# Application of Transfer Entropy Measure to Characterize Environmental Sounds in Urban and Wild Parks

**DOI:** 10.3390/s25041046

**Published:** 2025-02-10

**Authors:** Roberto Benocci, Giorgia Guagliumi, Andrea Potenza, Valentina Zaffaroni-Caorsi, H. Eduardo Roman, Giovanni Zambon

**Affiliations:** 1 Department of Earth and Environmental Sciences (DISAT), University of Milano-Bicocca, Piazza Della Scienza 1, 20126 Milano, Italy; g.guagliumi@campus.unimib.it (G.G.); a.potenza@campus.unimib.it (A.P.); valentina.zaffaronicaorsi@unimib.it (V.Z.-C.); giovanni.zambon@unimib.it (G.Z.); 2 Department of Physics, University of Milano-Bicocca, Piazza Della Scienza 3, 20126 Milano, Italy

**Keywords:** eco-acoustic indices, animal vocalization, urban park, transfer entropy measure

## Abstract

Anthropized green zones in urban areas and their surroundings develop complex soundscapes, characterized by the presence of multiple sound sources. This makes the interpretation of the sound environment challenging. To accurately distinguish between different sound components, a combination of selective analysis techniques is necessary. Urban parks are significant and interesting examples, where the interaction between anthropogenic and biophonic sound sources persists over broad temporal and spatial scales, making them important sites for evaluating local soundscape quality. In this work, we suggest that a transfer entropy measure (TEM) may more efficiently disentangle relevant information than traditional eco-acoustic indices. The two study areas were Parco Nord in Milan, Italy, and Ticino River Park, also in Italy. For Parco Nord, we used 3.5-h (1-min interval) recordings taken over an area of about 20 hectares, employing 16 sensors. For the Ticino River Park, we used 5-day (1 min plus 5 min pause) recordings taken over an area of approximately 10 hectares, using a smaller set of eight sensors. We calculated the classical eco-acoustic indices and selected two of them: the acoustic entropy (H) and the bio-acoustic index (BI), calculated for all sites with a 1 min time resolution obtained after a principal components analysis. For these time series, we studied the TEM of all sites in both directions, i.e., from one site to another and vice-versa, resulting in asymmetric transfer entropies depending on the location and period of the day. The results suggest the existence of a network of interconnections among sites characterized by strong bio-phonic activity, whereas the interconnection network is damped at sites close to sources of traffic noise. The TEM seems to be independent of the choice of eco-acoustic index time series, and therefore can be considered a robust index of sound quality in urban and wild park environments, providing additional structural insights complementing the traditional approach based on eco-acoustic indices. Specifically, TEM provides directional information about intersite sound connectivity in the area of study, enabling a nuanced understanding of the sound flows across varying anthropogenic and natural sound sources, which is not available using conventional methods.

## 1. Introduction

The complexity of soundscapes in urban and natural green areas is a key focus in the emerging field of soundscape ecology [1]. “Soundscape” refers to the interaction between a landscape and the mix of overlapping sounds from multiple sources.

These sounds often include biophony (animal vocalizations) [2], anthrophony (human activity), and geophony (natural sounds like wind or rain) [3]. These sound sources are typically differentiated through a visual inspection of sonograms. However, this method is impractical for long-term recordings, despite the development of automated techniques to identify biological sounds from spectrograms [4].

To address this challenge, ecoacoustic indices were introduced to simplify analysis. These indices focus on specific sound characteristics, such as pitch, modulation, saturation, and amplitude. Often referred to as eco-acoustic metrics, they are designed to assess the complexity and dynamics of acoustic environments, serving as proxies for various sound measures to evaluate environmental quality [5,6,7]. Unlike purely physical sound properties (e.g., sound pressure level or power spectrum density), ecoacoustic metrics provide additional insights. They have been widely used in ecological studies to explore diel cycles in tropical forests [8] and seasonal activities in both temperate and tropical habitats [9,10].

Research has shown that eco-acoustic indices can differentiate soundscapes across various habitats, shedding light on how intra- and inter-species communication may be disrupted. This highlights their potential for environmental research and sustainability efforts [11,12,13]. The standard approach to eco-acoustics has been extensively studied. For example, analyzing 60 h of recordings enabled researchers to identify distinct biophonic activities throughout the day using unsupervized statistical methods [14]. Additionally, a cross-correlation analysis of eco-acoustic indices from two sites in the same area provided insights into the underlying structure of park soundscapes [15].

Mapping the environment over large areas is now possible by using cost-effective audio-recorders, thus allowing the gradients of potentially disturbing sound sources to be discerned. For this reason, soundscape maps are becoming a widespread approach to evaluate sound quality in urban and natural green areas. Traffic noise maps have become popular to study large urban areas. They have been applied in large cities such as Madrid [16], Rome [17], Paris [18], Rotterdam [19], and Milan [20,21]. Thus, extending the concept of spatial representation to green areas has become an important staple across all fields of eco-acoustics and bio-acoustics. Indeed, overseeing the impact of urbanization over green areas by monitoring across space and time could provide useful information for conservation planning and improve the sustainability and biodiversity of green areas.

In this rapidly evolving context, we studied a new approach to investigating sounds present within two large green areas: an urban green area, the Parco Nord Milan, and a natural green area, within the Ticino River park (Milan). A preliminary study concerning the Parco Nord Milan has been reported recently [22]. The proposed method is essentially based on Shannon transfer entropy (TE) [23], here denoted as STE, which is a non-parametric measure of asymmetric information transfer between time series. In this context, it is useful to remind the reader that when we refer to information, we have in mind the concept of information theory, and that a key element in the latter is entropy. In his classic paper of 1948 (“A Mathematical Theory of Communication”), Claude Shannon introduces the concepts of both entropy and mutual information [24]. The former quantifies the average level of “information”, “surprise”, or “uncertainty” involved in the value of a random variable or outcome of a random process, whereas the latter, defined for two random variables, describes the measure of information in common between those variables, which can be used to describe their correlation.

Another important feature of a system consisting of more than one component is its structure; the latter can be obtained by measuring to what extent the individual components contribute to information production, and the rate of information exchange among them. This can be addressed on the basis of time series observations. One possibility is to quantify the overlap of the information content of two (sub)systems using mutual information (MI) [25]. However, since it contains neither dynamical nor directional information, MI cannot distinguish a potential direction, or the causality, of the coupling variables. Actually, by introducing a time delay in one of the observations an improvement was obtained in this respect, but the MI cannot distinguish whether the exchanged information is due to an input or represents the response to a common input signal.

A method sensitive to directional coupling between variables was explored using Granger causality (GC) [26]. However, since GC relies on correlation, it is limited to linear systems. To address this limitation, generalizations have been developed to quantify the nonlinear interactions between bivariate time series, introducing transfer entropy based on Shannon’s entropy [27]. This approach aims to capture both the dynamics and direction of information flow while incorporating some of the desirable properties of mutual information. Additionally, transfer entropy requires minimal assumptions about the underlying stochastic processes, enabling its broad applicability.

A different approach to calculating transfer entropy is also provided by Rényi transfer entropy (RTE) [28], which is able to weight the probabilities associated to tail events; that is, those that have a lower likelihood compared to the ones at the center of the distribution. In other words, RTE puts more weight on the tails when calculating the transfer entropy. This is particularly convenient when the tails of the distribution contain the most relevant information for the problem at hand.

TE approach has been applied across a wide range of fields. In neuroscience [29,30,31], it has been used to differentiate electroencephalography (EEG) data between healthy and epileptic individuals [32] and to study causal relationships, or effective connectivity, within the brain. This has revealed that brain dynamics are not fully explained by stimulus-response relationships alone, as a significant portion of brain activity is thought to be internally generated. Further studies consider the way information flows occur in the brain based on Shannon TE and other multivariate information measures [33,34,35,36,37].

Other applications include interpreting large-scale climate phenomena, such as the Antarctic Circumpolar Wave in the Southern Ocean [38], analyzing dynamic interactions and information flows using Shannon and Rènyi TE in stock markets [39,40,41,42,43,44], and detecting directional coupling changes in nonlinear biomedical time series, even with a limited number of observations and the presence of outliers [45]. Transfer entropy has also been employed to assess interactions between parasympathetic and sympathetic activities in the nervous system through heart rate variability [46] and to investigate the processes driving the dynamics of collective social phenomena [47]. In the following, we refer generically to TEM as either STE or RTE, depending on the context.

A novel approach like TEM is needed to capture the dynamics and specific directional relationships between sound sources across different sites. Traditional ecoacoustic indices provide valuable static measures of soundscape complexity, but cannot reveal how sounds interact or propagate through space. The application of TEM enables a refined analysis of acoustic information fluxes, providing deeper insights into ecological connectivity and the extent to which anthropogenic disturbances modulate natural soundscapes. While ecoacoustic indices effectively characterize sound diversity and intensity, they do not capture the causal and directional interactions between distinct acoustic sources, thus limiting their capacity to elucidate underlying ecological dynamics. They do not account for how disturbances, such as traffic noise, impact the spatial distribution of biophonic activity. TEM addresses this limitation by quantifying the potentially asymmetric transfer of acoustic information between sites, thereby elucidating the extent to which environmental and anthropogenic factors shape soundscape dynamics.

In this paper, we use TEM to extract spatio-temporal information on environment sounds, thus complementing the standard eco-acoustics approach. Indeed, the use of the latter in urban green areas may present some biases, thus making their interpretation not straightforward owing to the simultaneous presence of anthropogenic/technophonic sound sources [48]. The primary objective of this study is to assess how TEM can be effectively implemented to analyze soundscape dynamics. Specifically, we aim to investigate the directional flow of acoustic information in urban (Parco Nord Milan) and natural (Ticino River park) green areas to better understand ecological connectivity and the impact of anthropogenic noise. Thus, by combining TEM with eco-acoustic indices, we offer a novel perspective on soundscape quality, providing insights into how sound interactions shape ecological networks. This approach has practical implications for biodiversity conservation and soundscape management, especially in identifying areas where anthropogenic noise disrupts natural acoustic communications.

This study builds on previous research in Parco Nord Milan and the Ticino River park, which used soundscape and machine learning methods [22,49,50] to explore new spatial and temporal connections among recording sites in anthropized green areas. By combining TEM with traditional ecoacoustic indices, our work enhances the understanding of soundscape quality and highlights patterns of sound connectivity driven by biophonic activity. The findings identify the areas most impacted by anthropogenic noise, offering valuable insights for improving soundscape management and conservation strategies.

## 2. Materials and Methods

### 2.1. Area of Study

Both areas of study, Parco Nord Milan and Ticino River park, are located in the northern part of Italy. The first one is nested in the northern urban area of the city of Milan, whereas the latter is approximately 40 km southwest of the city in a much wilder area.

#### 2.1.1. Parco Nord Milan Area

**Figure 1:**
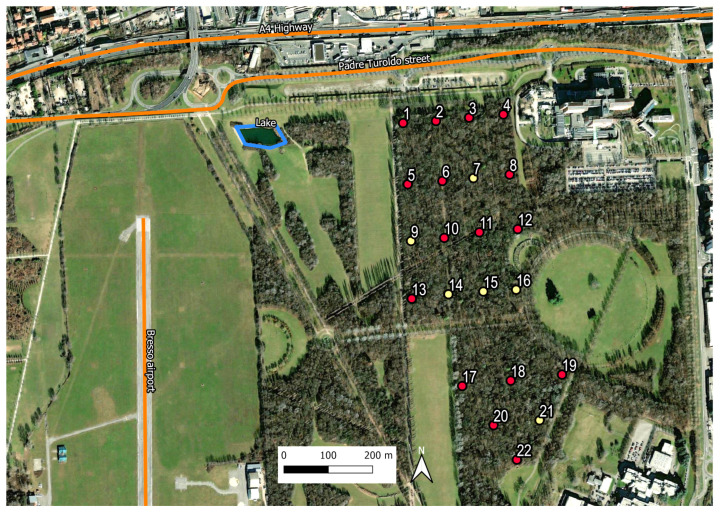
Parco Nord Milan (Italy) area of study (southern part of the A4 highway and the eastern part of the Bresso airport). Shown are the initially prepared N=22 measuring sites (circles); six sensors n°(7, 9, 14, 15, 16, 21) (yellow circles) had technical issues and were not included in the analysis. Thus, we obtained $N_{\mathrm{eff}}=16$ (red circles) effectively working sensors.

The area of study at the Parco Nord Milan spans a tree-covered parcel of over 20 hectares and is surrounded by congested roads (A4 highway and a thoroughfare road: Padre Turoldo street) and the runway of a small airport (Bresso airport). An artificial lake of approximately 300 m^2^ is also present at approximately 250 m from the edge of the bush (see Figure 1). Visitors mainly use this area for walking, playing sports, or recreational activities, as it is crossed by numerous paths. For this recording campaign, we selected, from a batch of 50 “very low-cost” recorders (SMT Security digital audio recorders with a 48 kHz sampling rate), N=22 audio recorders with similar frequency responses. Data were recorded during 25 May 2015, with the devices positioned on a grid, as shown in Figure 1. For each site, 3.5 h recordings were available (from 6:30 a.m. to 10:00 a.m.) in continuous mode. Details of the characterization and selection of the audio recording procedure are provided in [48]. As illustrated in Figure 1, six sensors (No. 7, 9, 14, 15, 16, 21, shown by the yellow circles) presented recording issues and were not used in this study. Thus, we used $N_{\mathrm{eff}}=16$ working sensors for the Parco Nord Milan.

#### 2.1.2. Ticino River Park Area

**Figure 2:**
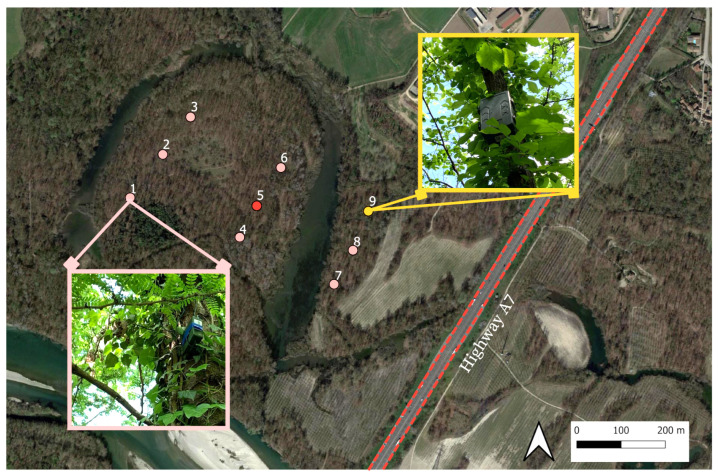
Area of study in Ticino River park (northern part of the river and western side of the highway A7). The nine initial measuring sites are indicated by the circles; sensor n°5 (red circle) was not used due to technical issues. Thus, $N_{\mathrm{eff}}=8$ in this case (orange circles). The SMM and SET recorders at sites 1 and 9 are shown (zoomed images within squares).

The area of study, located near the Moriano oxbow within the Ticino Valley Regional park, lies in the northwest part of Pavia province along the left bank of the Ticino River, encompassing approximately 39 hectares. This landscape includes sections of the floodplain, the Ticino valley depression, and adjacent low plains. A key anthropogenic influence is the A7 highway (“Autostrada dei Giovi”), which significantly impacts the area through noise pollution and habitat fragmentation (see Figure 2).

The area under study is encompassed within a Site of Community Importance (SCI) IT2080014, designated as “Basso Corso e Sponde del Ticino”. We selected nine locations (sites) distributed on a regular grid to represent different habitat types and we divided the area into two zones: a zone within the oxbow (sites 1–6), and a zone outside the oxbow (sites 7–9), closer to the highway (see Figure 2).

The recordings were made over two consecutive weeks (13–27 April 2022), following a protocol of 1 min recording followed by a 5 min pause. Eight (sites 1–8) song meter micro (SMM) recorders and one (site 9) soundscape explorer terrestrial (SET) recorder were deployed across the park. However, the device at site 5 was offline, leading to its exclusion from the analysis, i.e., $N_{\mathrm{eff}}=8$ in this case. Both types of recorders were set with a 48 kHz sampling rate.

#### 2.1.3. Eco-Acoustic Indices

To extract spectral characteristics from the audio recordings time series, we utilized eight ecoacoustic indices: Acoustic entropy (H) [5], acoustic complexity index (ACI) [6], acoustic diversity index (ADI) [24], acoustic evenness index (AEI) [51], normalized difference soundscape index (NDSI) [52], bioacoustic index (BI) [53], dynamic spectral centroid (DSC) [54], and zero crossing rate (ZCR) [55].

### 2.2. Statistics

Principal component analysis (PCA) is a multivariate technique that analyzes a dataset in which observations are described by several inter-correlated variables. In our case, we use eight variables, i.e., eight eco-acoustic indices for each site. The principal components are linear combinations of the original variables and are orthogonal to each other, meaning that correlated variables are merged together with different weights into new variables that are generally referred to as dimensions; these are indicated as Dim 1, Dim 2, etc.

PCA acts as a dimensionality-reduction method to lower the number of variables [56]. Each component Dim *i* has an associated weight, or variance $V_{i}$, with i = 1–8, within the PCA such that $\sum_{i=1}^{8}V_{i}=1$. Therefore, referring to the mean variance, $\bar{V}$, those components with $V_{i}\ll \bar{V}$ are assumed to be less relevant and can be discarded [57]. In the applications, we refer to the partial sum, $\sum_{i=1}^{i_{e}}V_{i}<1$, with $i_{e}<8$, as the cumulative explained variance (EV); this is expressed as a %. PCA thus allows us to work with only few eco-acoustic indices, those with the highest weights, $V_{i}$.

#### 2.2.1. Transfer Entropy Measure (TEM): STE and RTE

In the realm of information theory, Shannon entropy represents a benchmark measure of the information of a discrete random variable [24,58]. Shannon entropy quantifies the reduction in uncertainty obtained when measuring the value of the variable. Consider a discrete random variable, *J*, where different outcomes occur with different probabilities p(j). The average information, H(J), obtained per outcome is defined as follows:

(1) $$H\left(J\right)=-\sum_{j=1}^{n}p\left(j\right)\cdot \log_{2}p\left(j\right),$$

and is measured in bits. Here, *n* is the number of distinct outcomes associated with the probabilities p(j). Equation (1) may also be referred to as the average number of bits required to optimally encode independent draws of *J*.

In the case of two signals, *X* and *Y*, the former is said to cause the latter, when *Y* is better predicted, at future times, by the time series *X* at previous and present times, rather than by using the history *Y* alone [59,60]. As a measure of causality between *X* and *Y* ($X\equiv x_{t}$ and $Y\equiv y_{t}$), Schreiber [23] suggested to study deviations from a Markov behavior. In the latter, the probability of each event depends only on the state of the previous events,

(2) $$p\left(y_{t+1}|y_{t}^{n},x_{t}^{m}\right)=p\left(y_{t+1}|y_{t}^{n}\right),$$

where $x_{m}=\left(x_{1},\ldots ,x_{t-m+1}\right)$, $y_{n}=\left(y_{1},\ldots ,y_{t-n+1}\right)$, and p(.|.) represents the conditional probability distribution. Here, *m* and *n* represent the order or memory of generalized Markov processes *X* and *Y*, respectively. In this work, all TE values were calculated for cases m=1 and n=1. It is of note that Equation (2) is fully satisfied when the transition probabilities or dynamics of *Y* are independent of the past of *X*; that is, in the absence of causality from *X* to *Y*.

Following Schreiber, the presence of causality from *X* and *Y* can be detected by measuring departures from Equation (2) by using, e.g., the Kullback–Leibler divergence [61] between the two probability distributions [23]. This leads to the concept of Shannon transfer entropy (STE) from *X* to *Y*, defined as follows:

(3) $$\mathrm{STE}\left(X\to Y\right)=\sum_{y_{t+1},y_{t}^{n},x_{t}^{m}}p\left(y_{t+1},y_{t}^{n},x_{t}^{m}\right)\cdot \log_{2}\frac{p(y_{t+1}|y_{t}^{n},x_{t}^{m})}{p(y_{t+1}|y_{t}^{n})},$$

with STE (X→X)=0; the coefficient $p(y_{t+1},y_{t}^{n},x_{t}^{m})$ yields the probability of a transition to $y_{t+1}$ given the previous values $y_{t}^{n}$ and $x_{t}^{m}$. The advantage of using Equation (3) to quantify causality is that, in principle, it does not assume any particular model for the interaction between the two systems or signals, as reported in previous approaches [62]. This is particularly relevant in the presence of unknown non-linear interactions. As an additional remark, we note that the Kullback–Leibler divergence is closely related the so-called dissimilarity between time series [50], as briefly discussed in the Appendix A.

Transfer entropy can also be based on Rényi entropy [28,63]. The latter depends on a weighting parameter, *q*, and can be calculated as follows:

(4) $$H^{q}\left(J\right)=\frac{1}{1-q}\cdot \log_{2}\sum_{j=1}^{n}p^{q}\left(j\right),$$

with q>0. For q→1, the Rényi entropy converges to Shannon entropy, Equation (1). For 0<q<1, events with a lower probability have a higher weight, while for q>1, outcomes with a higher probability are favored [62]. Consequently, Rényi entropy allows for different areas of distribution to be emphasized, depending on *q*. The generalized *q*-moments, as in Equation (4), were used in the context of turbulence [64,65] and in many other non-linear systems with multifractal behavior [66,67]. The use of the Kullback–Leibler divergence was recently discussed in the context of offshore wind speeds [68]. The concept of multifractality remains to be investigated in the context of soundscapes.

Formally, applying the escort distribution, $\phi_{q}\left(j\right)=p^{q}\left(j\right)/\sum_{j}p^{q}\left(j\right)$ [69] and q>0 to normalize the weighted distributions, the Rényi transfer entropy (RTE) [63] can be written as follows:

(5) $$\mathrm{RTE}\left(X\to Y\right)=\frac{1}{1-q}\cdot \log_{2}\frac{\sum_{i}\phi_{q}\left(y_{t}^{n}\right)p^{q}\left(y_{t+1}|y_{t}^{n}\right)}{\sum_{i,j}\phi_{q}\left(y_{t}^{n},x_{t}^{m}\right)p^{q}\left(y_{t+1}|y_{t}^{n},x_{t}^{m}\right)}.$$

TEM is a robust and innovative tool for analyzing soundscapes as it quantifies the directional flow of information between time series without assuming linear relationships. This makes TEM particularly effective in capturing the complex, non-linear interactions that are typical of ecological systems. Unlike correlation-based methods, TEM identifies asymmetrical and dynamic dependencies, allowing for the detection of possible causal influences between sound sources, which is essential for understanding how natural and anthropogenic sounds interact within an environment.

#### 2.2.2. Computation

We adopted R-software, version 4.5.0, for calculating ecoacoustic indices, PCA, and TE matrices, using the following packages: Stats [70] for PCA, Soundecology [71] for ecoacoustic indices, Seewave [72] for audio analysis, and RTransferEntropy [62] for TEs.

### 2.3. Aural Survey

Quantification of the bio-, techno- and geo-phonies present at a given site requires an aural survey. This means that a single expert listens all the recordings, labelling the audio files according to the categories illustrated in Table 1. The category “Singing activity” was expressed in terms of % of singing time in each 1 min recording; for instance, 25% represents a time span of 15 s.

### 2.4. Analysis Scheme: TEM Direction Flow

**Figure 3:**
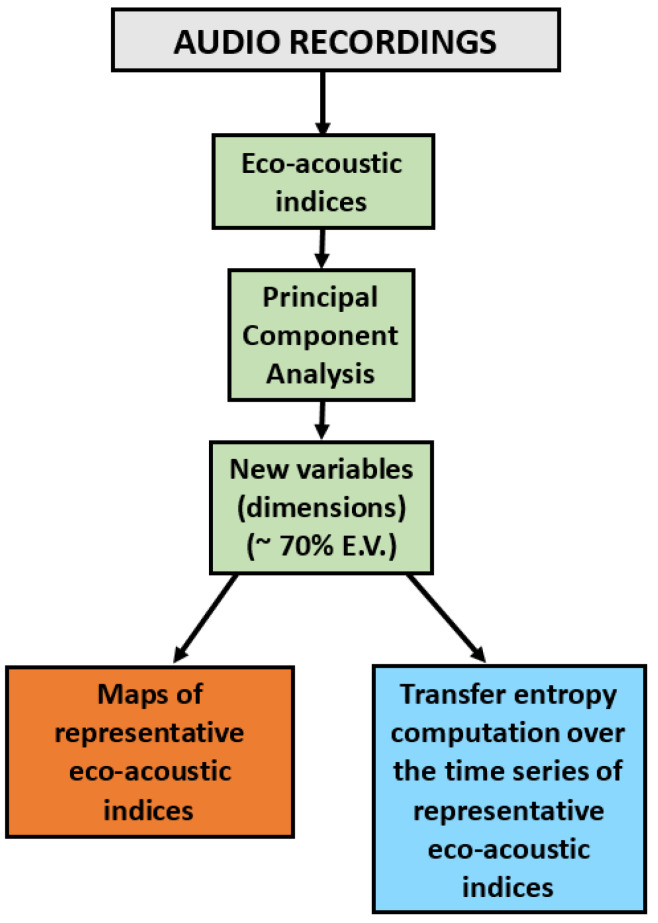
Flow diagram of the complete analysis used to obtain the TEMs. The abbreviation E.V. stands for the cumulative explained variance (see Section 2.2).

The analysis scheme adopted here is displayed in Figure 3, where the flow chart illustrates the way in which we calculate the eco-acoustic indices (see Section 2.1.3) from the audio recordings at each measuring site. Then, we used the PCA to group correlated variables together, thus reducing the number of original variables and obtained the new variables (dimensions Dim 1, Dim 2, etc.) as a combination of the original ones.

For each dimension, we selected a representative index, in keeping with the need to simplify the interpretations of the results, and calculated the TEMs from the corresponding time series. The calculation was performed for each site, represented by its time series *X*, and all the other sites, with time series denoted as *Y*, to obtain TEM (X→Y) and TEM (Y→X), according to Equation (3). This calculation allows us to determine possible asymmetries in the information flow direction. The presence of a dominant direction of information flow can be inferred by calculating the difference, DTEM, between the TEMs in both directions:

(6) DTEM=TEM(X→Y)−TEM(Y→X).

The direction of the TEM flow between two sites is taken from X→Y if DTEM > 0, or from Y→X otherwise, and is represented by an arrow connecting the two sites on a graph. The difference between TEMs is denoted as DSTE and DRTE for the Shannon and Rényi TE, respectively.

DTEM complements traditional eco-acoustic indices, like H and BI, by adding a dynamics and directional dimension to the analysis. While H and BI effectively describe the complexity and intensity of soundscapes, they provide static and non-directional insights. DTEM enhances these measures by revealing how acoustic information flows between sites, highlighting areas where biophonic activity is dominant or where anthropogenic noise disrupts natural communication. This combined approach offers a more comprehensive understanding of a soundscape’s structure and dynamics. The network of interconnections derived from DTEM provides a visual and quantitative illustration of the directional flow of acoustic information across different sites. Indeed, understanding these patterns is crucial for soundscape management, allowing for the identification of critical areas that require conservation efforts or noise-mitigation strategies to preserve ecological integrity and biodiversity.

## 3. Results

**Figure 4:**
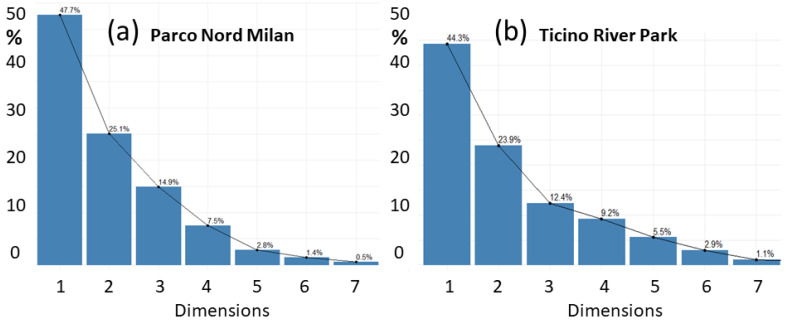
Explained variance [%] vs. dimensions for (**a**) PNM, EV = (47.7, 25.1, 14.9, 7.5, 2.8, 1.4, 0.5)%; (**b**) TRP, EV = (44.3, 23.9, 12.4, 9.2, 5.5, 2.9, 1.1)%. Since, in most cases, Dim 8 presents values that are too small, we discarded it from the plots.

As described in Section 2.4, the analysis of audio recordings collected over the two areas of study started with the calculation of the ecoacoustic indices reported in Section 2.1.3. The ecoacoustic indices were integrated over a 1 min temporal window. For Parco Nord Milan, we obtained a 3.5 h continuous recording, yielding a time series of 210 elements long. For the Ticino River park, we obtained 1 min recordings followed by a 5 min pause over a period of 2 weeks, yielding a series of 3360 elements. The PCA was applied separately to both time series, thus reducing the number of variables and obtaining insights into their relevance. The explained variances, as a function of the number of dimensions (see Section 2.2), are shown in Figure 4a for Parco Nord Milan, and in Figure 4b for Ticino River park.

In keeping with the smallest set of dimensions and largest possible variance, we selected the first two dimensions out of the eight total. Dimensions Dim 1 and Dim 2 have a cumulative explained variance of V=V1+V2, yielding V=(47.7+25.1)=72.8% for Parco Nord Milan (Figure 4a) and V=(44.3+23.9)=68.2% for Ticino River park (Figure 4b). This choice was considered suitable for our purposes.

**Figure 5:**
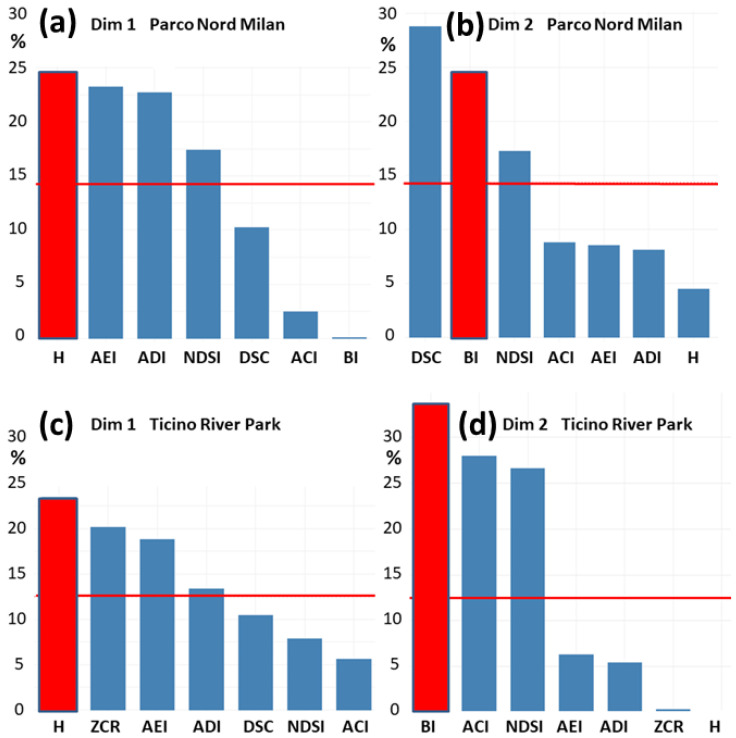
Fractional contributions [%] of indices to dimensions for: (**a**) Dim 1 PNM and (**b**) Dim 2 PNM; (**c**) Dim 1 TRP and (**d**) Dim 2 TRP. The horizontal red lines denote the mean explained variance for each dimension. The vertical red bars denote the indices—H for Dim 1 and BI for Dim 2—chosen as representative for each dimension.

The contributions of the indices to Dim 1 and Dim 2 in Parco Nord Milan are shown in Figure 5a,b, and in Ticino River park in Figure 5c,d. For Parco Nord Milan, the contributions to the first dimension, Dim 1 (Figure 5a), are almost equally distributed over the three indices, H, AEI, and ADI, with a weight of about (22–24)% each and with a slightly lower value for NDSI (∼17%). The second dimension, Dim 2 (Figure 5b), is largely shown by two indices, i.e., DSC (28%) and BI (24%).

For Ticino River park, Dim 1 (Figure 5c) is almost equally distributed over the three indices, H (23%), and ZCR ∼ AEI (17–20)%. The second dimension, Dim 2 (Figure 5d), is largely shown by three indices, i.e., BI (34%) and ACI ∼ NDSI (27%).

To describe the behavior of the two dimensions, Dim 1 and Dim 2, that are uncorrelated by, we took H as the representative for Dim 1, and BI for Dim 2 (see the red bars in Figure 5). As a reminder, the acoustic entropy, H, provides information about the homogeneity of frequency in both the time and frequency domains, whereas the bioacoustic index, BI, provides information about the area below the mean spectrum in the frequency range, which is typically occupied by biophonies (2000–8000 Hz). We note that in [22], we made a slightly different choice. This does not affect our present conclusions.

### 3.1. Parco Nord Milan: TEM Results

**Figure 6:**
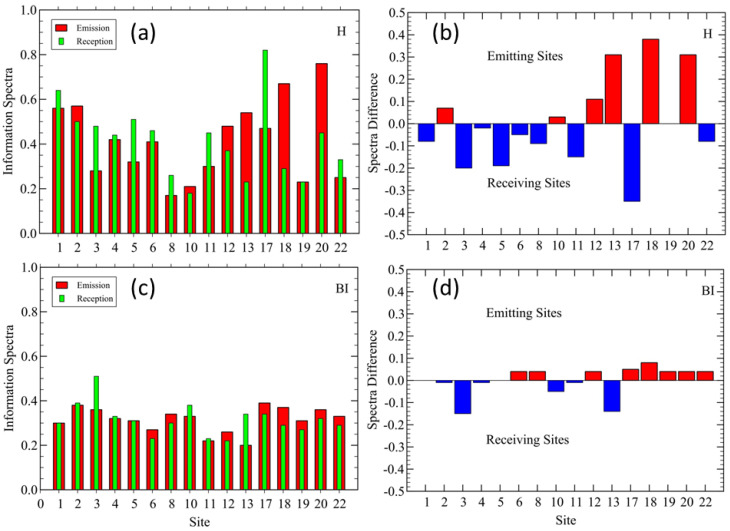
Parco Nord Milan. (**a**) Total emissions and reception of STE at each site and (**b**) difference between total emission and reception spectra, from (**a**), for index H. (**c**) Same as in (**a**), and (**d**) same as in (**b**), for index BI. In (**a**,**c**), the red bars represent the total STE from each site to all others sites, while the green bars represent the total STE received at each site. In (**b**,**d**), the predominantly emitting sites are represented by the red bars (bio-phonic ‘hot’ sites), while the predominantly receiving ones are represented by the blue bars (bio-phonic ‘cold’ sites).

Before discussing the details of the TEMs between sites, it is illustrative to look at their mean local values. The idea was to evaluate the total TEM emitted from *X*, $\sum_{Y}\mathrm{TEM}\left(X\to Y\right)$ and received at *X*, $\sum_{Y}\mathrm{TEM}\left(Y\to X\right)$. The results are shown in Figure 6a,c for indices H and BI, respectively. In Figure 6b,d, we show the difference between the emission and reception spectra at each site to highlight their different locations in the network. The predominantly emitting sites were found in the interior of the park, as expected.

**Figure 7:**
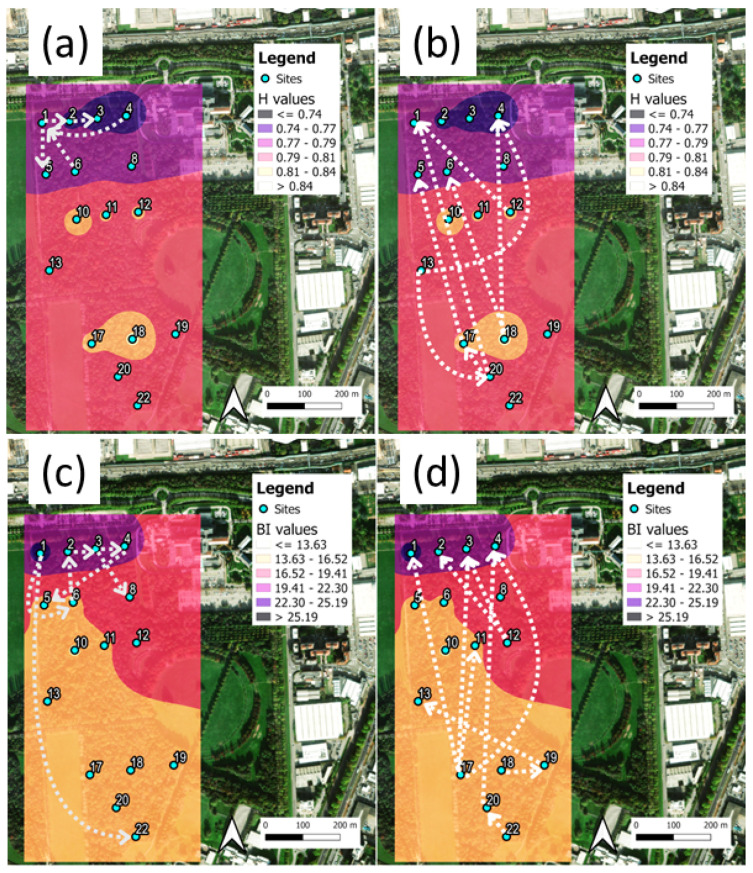
Parco Nord Milan heat maps and main Shannon TE flows, DSTE. (**a**) Mean values of H and local interconnections at small spatial distances between sites (DSTE arrows). (**b**) Mean values of H and interconnections over longer distances between sites. The range of mean values are represented by different colors; the color scale is reported in the insets. (**c**) Same as in (**a**), for index BI (we added a long-range arrow 1 → 22). (**d**) Same as in (**b**) for index BI. In all cases, the DSTE direction is represented by arrows and only values above the 95th percentile are shown, for simplicity.

The eco-acoustics index heat maps are shown in Figure 7 for the H and BI indices, where, in addition, the Shannon TE flows and DSTE > 95th percentile are indicated by arrows. The full data for both the STE and DSTE (index H) at Parco Nord Milan are reported in Appendix B. The complete results, including both H and BI, at Parco Nord Milan and Ticino River park are available in the Supplementary Information file.

In Figure 7a,b, the heat map for the H index shows higher values in the interior of the park, thus indicating the presence of birdsong and, in addition, the low frequencies coming from the multiple sources of very congested traffic noise, located at the northern side of the park. Also, the BI index (Figure 7c,d) is biased by these techno-phonic noise sources, which lead to overestimations of the index value. More interestingly, the representation of TEM over the map presents two different patterns: the first is a pattern of interconnections among sites that are sitting close to the traffic noise source, characterized by a short spatial scale (Figure 7a,c), and the second originates from sites sitting inside the park characterized by interconnections between sites over a larger spatial scale (Figure 7b,d).

**Figure 8:**
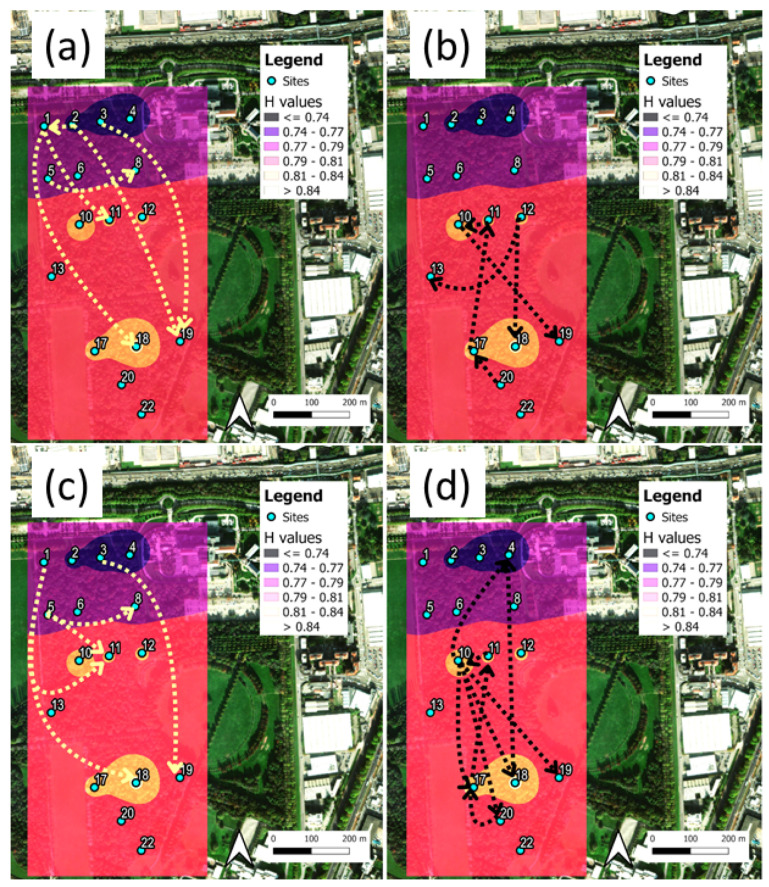
Parco Nord Milan heat maps and main Rényi TE flows, DRTE, for index H. (**a**) Mean values of H for q=0.1 and DRTE arrows originating from northern sites or the edges of the park. (**b**) Mean values of H for q=0.1 and DRTE arrows originating from central–southern sites, located in the interior of the park. (**c**) Same as in (**a**) for q=0.5. (**d**) Same as in (**b**) for q=0.5. In all cases, we have shown values DRTE > 95th percentile.

**Figure 9:**
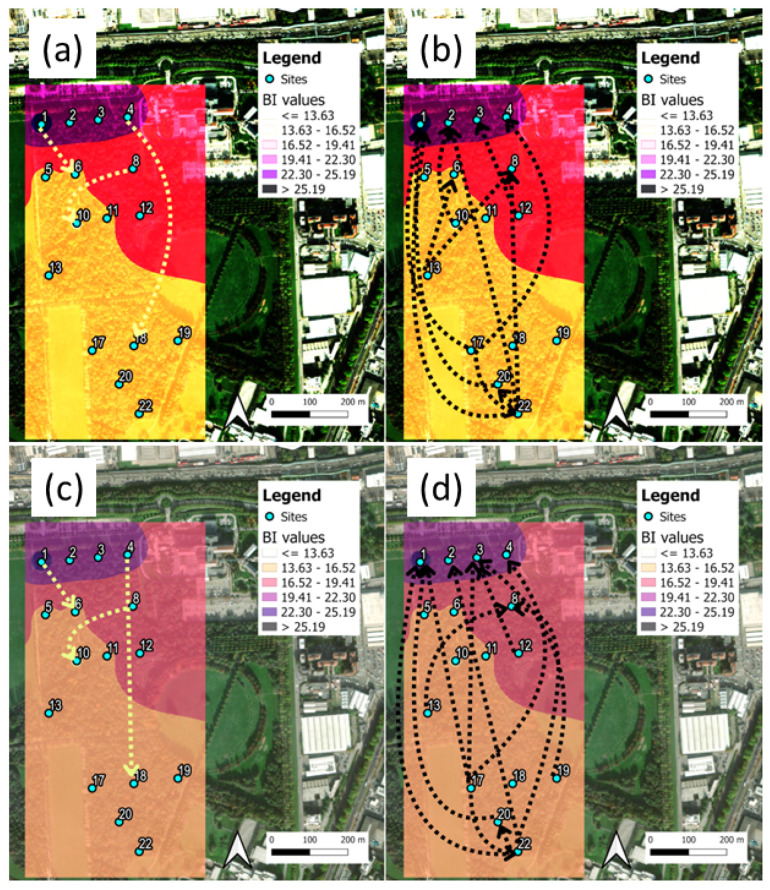
Parco Nord Milan heat maps and main Rényi TE flows, DRTE, for index BI. (**a**) Mean values of BI for q=0.1 and DRTE arrows originating from northern sites or edges of the park. (**b**) Mean values of BI for q=0.1 and DRTE arrows originating from central–southern sites, located in the interior of the park. (**c**) Same as in (**a**) for q=0.5. (**d**) Same as in (**b**) for q=0.5. In all cases, we have shown values DRTE > 95th percentile.

Figure 8 shows the mean H index map for the Rényi transfer entropy, and the main flows, DRTE, for cases q=0.1 (Figure 8a,b) and q=0.5 (Figure 8c,d). The cases shown in (a,c) and (b,d) represent two different DRTE interconnection patterns originating from the northern and central–southern sites, respectively. In this case, both the q=0.1 and q=0.5 parameters show a network of interconnections between DRTEs that seem to converge toward the park interior, supplemented by the homogenization of the spatial scales. Figure 9 reports the corresponding results for the BI index. We note that for both q=0.1 and q=0.5, the results of the interconnection patterns are similar to those obtained for the DSTE (Figure 7) for the sites sitting inside the park, while the interconnections originating at the northern border of the park have almost vanished.

### 3.2. Ticino River Park: TEM Results

For the Ticino River park, we split the results into three time intervals: dawn (from 05:00 to 08:00), day (from 08:00 to 18:00), and night (from 18:00 to 05:00). This choice was motivated by the possibility of observing eventual changes in the analysis caused by the presence of different sound sources during the various periods of the day.

**Figure 10:**
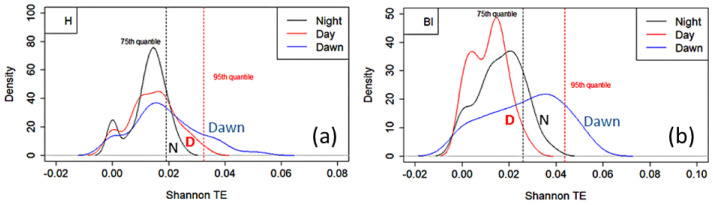
Ticino River park. Probability density distribution of Shannon transfer entropy vs. STE calculated for the representative indices (**a**) H and (**b**) BI. The STEs were computed from each site to all the other ones. In the plot, the two vertical dashed lines represent the 75th (black dashed line) and 95th (red dashed line) percentiles. The STE values were split into night (black line), day (red line) and dawn (blue line) periods.

Figure 10 illustrates the probability density distribution of the Shannon TE calculated for the two representative indices: H and BI. The TEMs were computed from each site to all the other ones. In the plot, the two vertical dashed lines represent the 75th (black dashed line) and 95th percentiles (red dashed line) of all the TEM values. During the dawn period, transfer entropy values were notably higher across all sites, reflecting the peak in biophonic activity typically observed during early morning hours, when avian species are most vocal.

**Figure 11:**
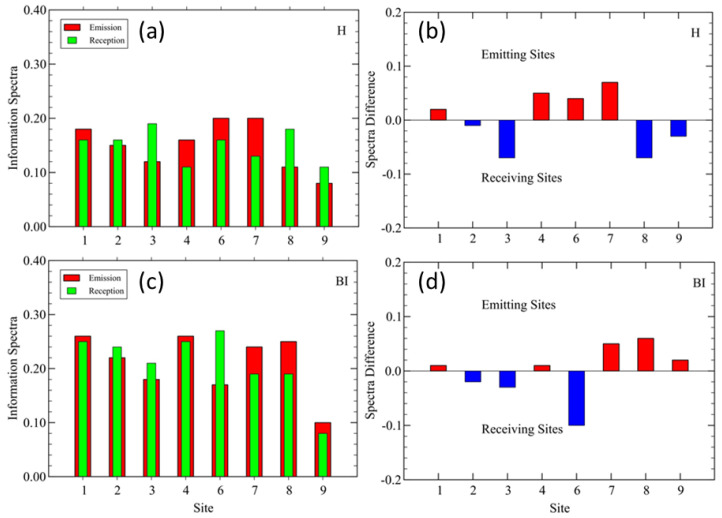
Ticino River park: (**a**) Total emission and reception of Shannon TE (STE) at each site, evaluated at dawn. (**b**) Difference between total emission and reception spectra, from (**a**), for index H. (**c**) Same as in (**a**), for index BI. (**d**) Same as in (**b**), for index BI.

The mean local values for the STE and their differences at each site, evaluated at dawn, are shown in Figure 11. The predominant emitting sites were located inside the park.

**Figure 12:**
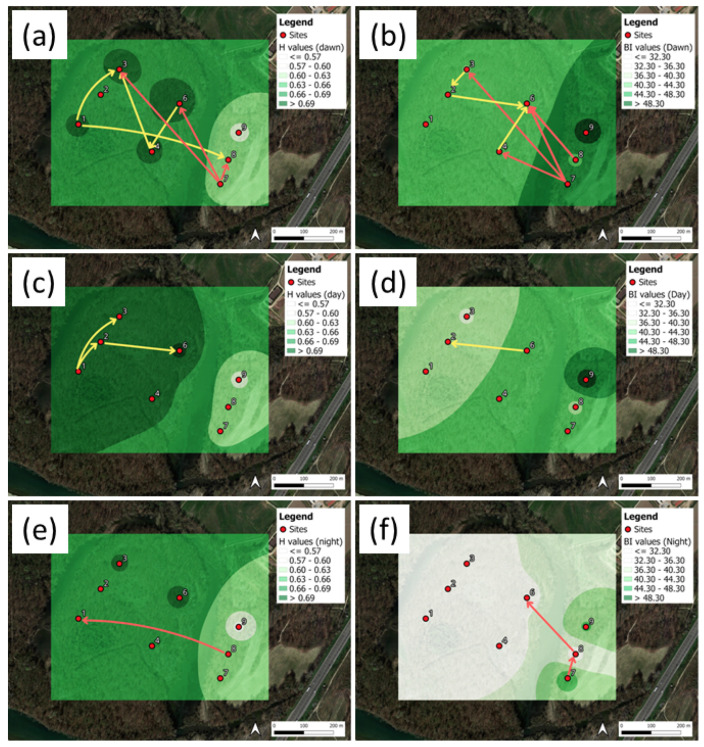
Ticino River park. Shannon TE flows, DSTE, for the indices (**a**,**c**,**e**) H, and (**b**,**d**,**f**) BI. The plots correspond to the day periods: (**a**,**b**) dawn; (**c**,**d**) day; (**e**,**f**) night. The DSTE are represented by the arrows, corresponding to DSTE > 95th percentile.

The TRP heat maps for the mean indices are shown in Figure 12a,c,e for H, and in Figure 12b,d,f for BI. The mean index values were averaged over the three daytime intervals: dawn (a,b), day (c,d), and night (e,f). Shannon flows, DSTE > 95th percentile, are shown by the arrows (see the Supplementary Information file for the full numerical data).

The maps of the acoustic entropy, H (Figure 12a,c,e), present higher mean values for the day period, followed by the dawn and night periods. As a matter of fact, higher H values mean higher spectrum homogeneity in both the time and frequency domains. Therefore, we argue that a change in the sound source composition might have an influence on the index computation. Actually, at dawn, we expect the presence of an intense bio-phonic sound source, which is slightly reduced during the day in concomitance with the presence of traffic noise. The contribution of these two sources make the overall spectrum more uniform in both the time and frequency domains. During the night, both components are significantly reduced.

Another observation regards the lower H values at sites 7, 8, and 9. For the latter, we found two concurring causes of this result. Firstly, the presence of the nearby A7 highway and, secondly, their position at the bush border with a lawn parcel. A more interesting result can be observed by looking at the TEM representations. Indeed, according to the expection that more bio-phonic activities are present at dawn, we found a network of interconnections among sites that is much higher than during the day and night periods. More specifically, during the day, we could still observe interconnections in the interior of the area, whereas during the night, the interconnections tended to fade.

A similar behavior was observed for the bioacoustic index, BI (Figure 12b,d,f). BI is, by definition, more correlated with both the biophonic activities and levels. This was observed at dawn, where on average, higher BI values than during the day and night periods are obtained. For BI, we found an opposite gradient with respect to H, suggesting, firstly, a possible contribution of the high frequencies associated with traffic noise, and secondly, the vicinity of the noise source. For instance, if a bird was singing very close to a microphone, we would obtain higher values for this index than another, more distant, microphone recording the same activity. This picture is also consistent with H and BI being anticorrelated.

**Figure 13:**
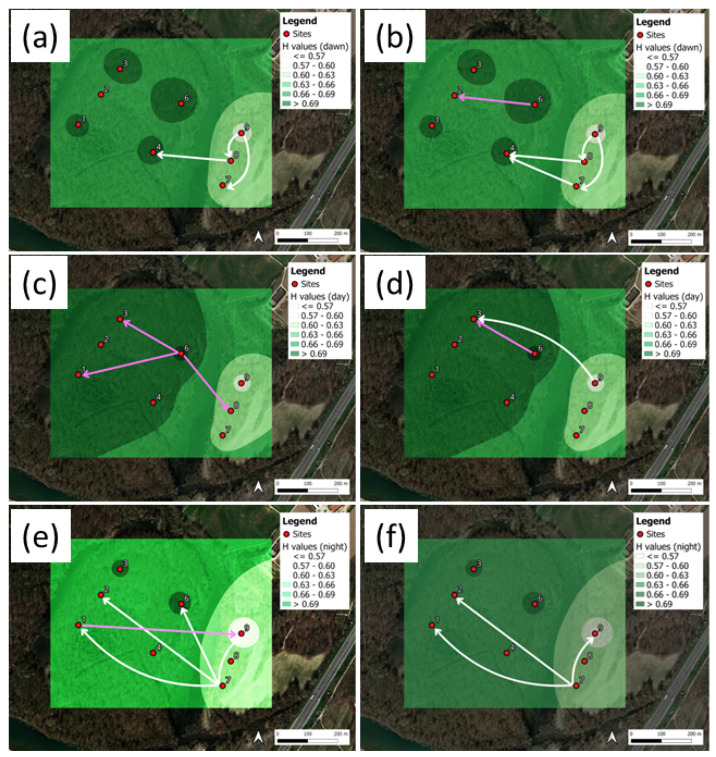
Ticino River park. Rényi flows, DRTE, for the H index: (**a**,**c**,**e**) q=0.1, (**b**,**d**,**f**) q=0.5. The plots correspond to the periods of (**a**,**b**) dawn, (**c**,**d**) day, and (**e**,**f**) night. The DRTEs are represented by the arrows, corresponding to DRTE > 95th percentile.

**Figure 14:**
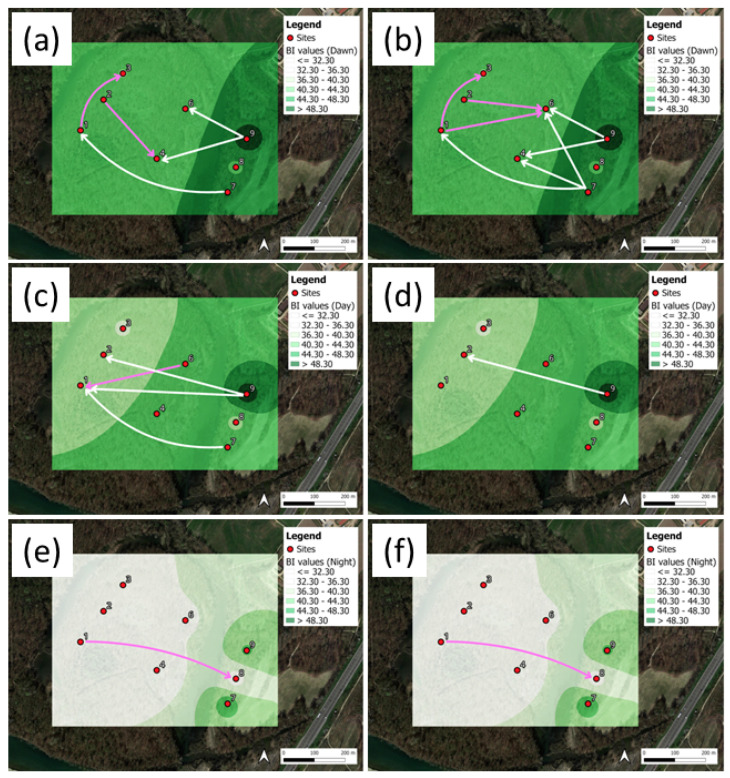
Ticino River park. Rényi flows, DRTE, for the index BI: (**a**,**c**,**e**) q=0.1, (**b**,**d**,**f**) q=0.5. The plots correspond to the periods of (**a**,**b**) dawn, (**c**,**d**) day, and (**e**,**f**) night. The DRTEs are represented by the arrows, corresponding to DRTE > 95th percentile.

Finally, the Rényi TE results are presented in Figure 13 for H, and in Figure 14 for BI, and the corresponding mean index maps with DRTE values above the 95th percentile.

Parts (a, c, e) of each figure refer to the weighting parameter q=0.1, whereas parts (b, d, f) refer to q=0.5. The latter provide high and moderate weights to (infrequent) tail observations, respectively. Regarding Figure 13, the DRTEs calculated for the H index do not present specific patterns, and the interconnection density among sites is lower than that observed for the DSTEs. The majority of interconnections were directed to the park’s interior, and no significant differences were observed between both *q* cases.

The DRTE results for BI (Figure 14) show a network of interconnections similar to the DSTE pattern, but in this case we can also observe a transfer of information from the periphery toward the interior of the bush. Cases q=0.1 and q=0.5 yielded the same pattern, with the exception of day period, for which q=0.1 shows more complex interconnections.

## 4. Discussion

Over the past decade, the methods used to evaluate information transfer across various fields have increasingly relied on the nonlinear measure of transfer entropy to estimate connectivity efficiently [73,74]. This has proven particularly valuable in neuroscience [75], where the brain is viewed as a fully integrated system, with regions that continuously exchange information in a dynamic and reciprocal manner [76,77]. A key motivation for the present study lies in the analogy between the anatomical connectivity within brain regions and the soundscapes produced by biophonies in nature, which can be conceptualized as a dynamic and complex system.

An important point when studying complex soundscapes over extended areas, where sound sources of different origins coexist, is that the use of “standard” ecoacoustic indices may introduce uncontrolled biases for multiple reasons. Firstly, the use of pocket digital recorders with different trademarks could lead to inevitable differences in the frequency responses. As a consequence, the use of different recorders to map extended areas would easily introduce misleading interpretations of the soundscape components, and therefore misleading habitat assessments [78]. Secondly, based on the information extracted by the selected ecoacoustic indices from the audio recordings, we may, in some cases, obtain distorted conclusions.

Indeed, by looking, for instance, at Figure 7 and Figure 12, we can observe a coherent distribution of the indices over both areas of study, with higher H index values obtained in the interior of the parks, away from traffic noise. Thus, both the contribution of biophonies and the low-frequency component of the traffic noise source (shown in the propagation and diffraction attenuation) make the local spectrum more homogeneous in terms of frequency content and more homogenous over time. This is likely true for the Ticino River park during the day period (Figure 12, H index), but it is less immediately clear for the dawn period. Indeed, for the latter, the prevalence of biophonies present inside the park, which are more noticeable than the negligible nightly road traffic noise, changes the interpretation.

The explanation for the BI index in complex soundscapes is less obvious. At Ticino River park, the BI index, predominantly influenced by biophonic activities, exhibits higher average values at dawn compared to the day and night periods, highlighting the peak in biological activity during the early morning hours. However, if we look at its distribution over the two areas of study, we observe, in both cases, higher values for those sites sitting close to the sources of traffic noise. This result might be due to high-frequency components (>2000 Hz) that are picked up in the index calculation and produced by the traffic noise source. In view of this issue, we consider introducing an index directly linked to the natural interconnection network, which is borrowed from the approach used to study living organisms, and could represent a step forward in describing complex sound environments.

For this reason, we explored two variants of TEM: STE and RTE. The main difference is that Rényi entropy emphasizes different areas of a distribution, depending on the weighting parameter *q*. When parameter *q* is lower, the more rare observations at the tail of the distributions are deemed important in the information flow process. As RTE converges to STE for q→1, the latter places more weight on data around the centre of the distribution.

A comparative analysis of Figure 7 and Figure 12 suggests that STE patterns exhibit independence from specific ecoacoustic indices, although H and BI are anticorrelated to each other. The RTE analysis reveals that information transfer triggered by tail events predominantly flows from the park’s edges toward the interior, suggesting a gradient in acoustic connectivity potentially driven by biophonic activity and noise diffusion patterns. However, a distinction between H and BI is in place. For H, fewer interconnections are observed, especially at the Ticino River park, whereas, for BI, the RTE provides a more complex network of interconnections. Thus, this observation suggests that when RTE is applied to an index directly correlated with biophonic activity, it preserves its original meaning.

The spatial analysis of the H and BI indices across sites revealed distinct variations in connectivity patterns due to the proximity to anthropogenic noise sources. To be noted is that sites adjacent to the A7 (Ticino River park) and A4 (Parco Nord Milan) motorways exhibited reduced bioacoustic connectivity, likely due to the masking effects of traffic noise on biophonic activity. In contrast, sites located further inside the park displayed increased bioacoustic connectivity, especially at dawn, when biophonic activity peaks. These results indicate that anthropogenic noise may interfere with natural communication networks, attenuating biophonic signal transmission and compromising ecological connectivity, a fundamental component of species interactions within soundscapes.

An important feature can be obtained by considering the density distribution of arrows (interconnections) per site and the spatial scale associated with the arrow length (normalized distance among interconnections, $0<d_{n}<1$). The latter, also denoted as the normalized distance index (NDI), is calculated as follows:

(7) $$d_{n}=\frac{1}{\bar{n}}\sum_{i=1}^{\bar{n}}\frac{d_{i}}{d_{\max}},$$

where $\bar{n}$ represents the overall interactions, i.e., total number of arrows (see e.g., examples in Figure 7, Figure 8 and Figure 9,Figure 12, Figure 13 and Figure 14), among sites, and $d_{\max}$ represents the maximum distance between interconnections.

**Figure 15:**
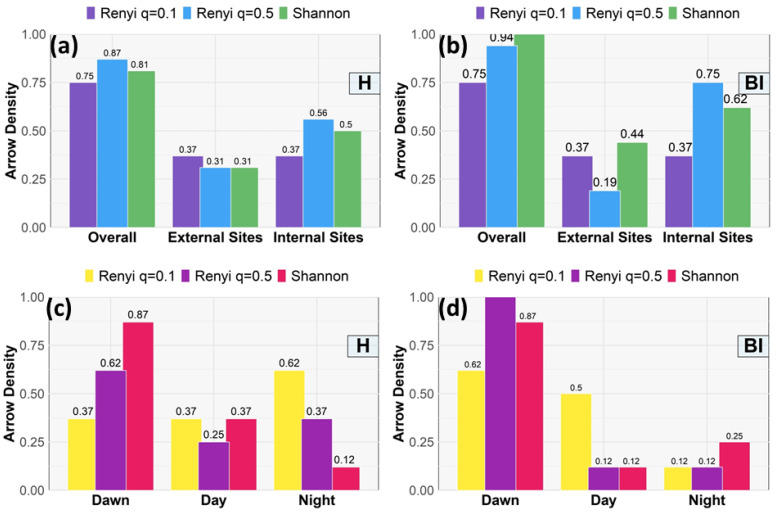
Arrow density. (**a**) PNM index H: overall sites (left bars), external sites (1, 2, 3, 4, 5, 6 ,8) (central bars), and internal sites (17, 18, 19, 20, 22) (right bars), vs. site type. The bars include the Rényi results (q=0.1, violet bars, q=0.5, blue bars) and Shannon results (green bars). (**b**) Same as in (**a**) for index BI. (**c**) TRP index H for Rényi (q=0.1, yellow bars, q=0.5, violet bars) and Shannon results (red bars), vs. time interval. The latter are separated into three periods: dawn (left bars), day (central bars), and night (right bars). (**d**) Same as in (**c**) for index BI.

The arrow densities are illustrated in Figure 15a,b for Parco Nord Milan and in Figure 15c,d for Ticino River park. For Parco Nord Milan, the sites are classified according to their location in the park, distinguishing internal sites (17, 18, 19, 20, 22) from external ones (1, 2, 3, 4, 5, 6 ,8). Due to the larger area involved in the PNM and the shorter recordings, we decided to plot the arrow densities for the external and internal sites (see Figure 15a,b). We observed an increase in arrow densities for sites in the interior of the park for both indices, although BI appears to be higher. In this case, all considered TEMs showed an increment. For the Ticino River park, Figure 15c,d, we considered the three time periods of dawn, day, and night instead (cf. Section 3.2), where the arrow densities show a clear increase at dawn, especially in the case of STE, for both the H and BI indices, while the RTE does not contribute to H but is very active for BI, meaning that the interconnections among sites are also created by occasional events.

More specifically, the analysis of arrow density across the Parco Nord Milan and Ticino River park provides a quantifiable measure of interconnectivity influenced by spatial and temporal sound dynamics. At Parco Nord Milan, the distinction between internal and external sites reveals a notable gradient in connectivity. External sites, influenced by anthropogenic noise sources such as traffic, show reduced arrow density, indicating limited information transfer. In contrast, internal sites, shielded from direct noise intrusion, exhibit higher arrow density, suggesting a more cohesive acoustic environment that is conducive to biophonic interactions. This spatial variation underscores the disruptive impact of techno-phonic noise on ecological communication networks [12].

At Ticino River park, the dawn showed the highest density of interconnections for both STE and RTE, reflecting the heightened biophonic activity typical of early morning hours. This result aligns with previous studies that identified dawn as a critical period for avian vocalizations, which dominate the acoustic landscape [5]. Conversely, during the night, the diminished density highlights the reduced ecological connectivity, likely due to the absence of significant biophonic signals.

**Figure 16:**
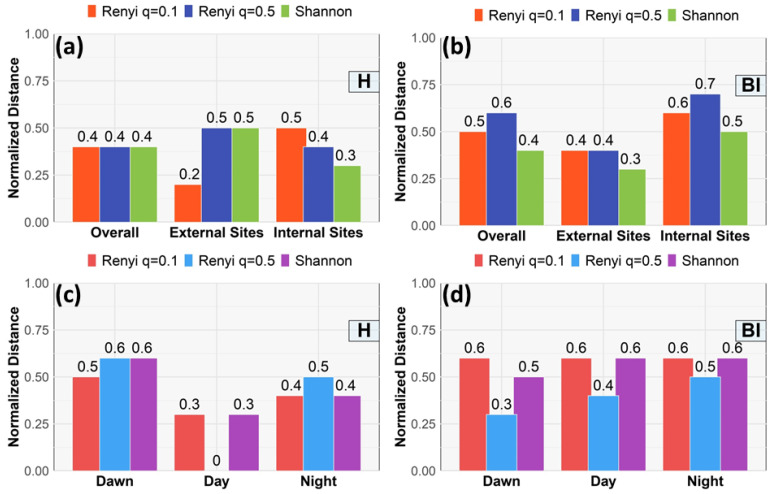
Normalized distance index, $d_{n}$. (**a**) Parco Nord Milan index H, $d_{n}$ vs. site type. NDI for: overall sites (left bars), from external sites (central bars) where the anthopized sources are located and from internal park sites (right bars). The bars include Rényi results (q=0.1, red bars, q=0.5, black bars) and Shannon results (green bars). (**b**) Same as in (**a**) for index BI. (**c**) Ticino River park index H, $d_{n}$ vs. time interval. NDI for Rényi (q=0.1, red bars, q=0.5, blue bars) and Shannon results (violet bars). (**d**) Same as in (**c**) for index BI.

The normalized distance index, NDI (Equation (7)), is illustrated in Figure 16a,b for Parco Nord Milan and in Figure 16c,d for the Ticino River park. For Parco Nord Milan, higher values of $d_{n}$ indicate more information transfer over relatively long distances. This may suggest more widespread human influence or more effective sound communication between distant sites. On the contrary, lower values of $d_{n}$ may suggest limited information transfer over shorter distances, which may reflect acoustic fragmentation or a localized sound environment.

For the Ticino River park, dawn showed the highest $d_{n}$ values, suggesting extensive spatial connectivity driven by biophonic activity. This reflects the capacity of acoustic signals, particularly avian vocalizations, to propagate over larger distances in the absence of competing noise sources [6]. The gradual reduction in $d_{n}$ values during the day and night highlights the role of anthropogenic noise and reduced biological activity in fragmenting the soundscape.

The normalized distance suggests additional insights into the spatial scale of acoustic interconnections within the studied areas. At PNM, internal sites exhibit higher $d_{n}$ values compared to external sites, indicating that protected areas within the park maintain more extensive acoustic connectivity. External sites, which are closer to noise sources, show lower $d_{n}$ values, suggesting the presence of localized sound zones fragmented by high traffic noise, consistent with acoustic masking theories, where anthropogenic noise interferes with the perception and propagation of biophonic signals [79]. Anthropogenic noise thus exerts a dual effect: reducing the spatial scale of natural acoustic connectivity, and creating localized sound zones dominated by technophonic sources. This observation reinforces the importance that spatial and temporal dynamics play in shaping acoustic interconnectivity. The clear gradient observed between internal and external sites at PNM stresses the potential of TEM as a robust tool. These results contribute to a growing body of evidence emphasizing the need for targeted noise-mitigation strategies to preserve the ecological functions in urban green spaces.

### Audio Surveys for Parco Nord Milan and Ticino River Park

**Figure 17:**
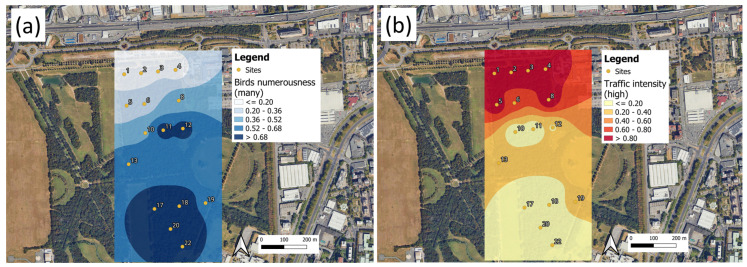
Parco Nord Milan: mean values of soundscape categories as perceived by the acoustic expert. (**a**) Number of birds (many birds singing); (**b**) traffic intensity (high traffic intensity). The reported values represent the fraction of recordings presenting the characteristics shown in the legend. The actual values represented in the plot are reported in Table 2.

**Figure 18:**
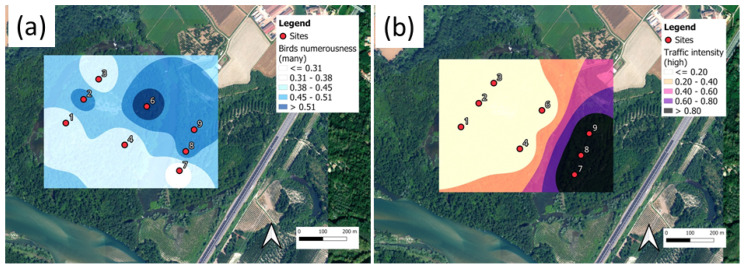
Ticino River park: mean values of soundscape categories as perceived by the acoustic expert. (**a**) Number of birds (many birds singing); (**b**) traffic intensity (high traffic intensity). The reported values represent the fraction of recordings presenting the characteristics shown in the legend. The actual values represented in the plot are reported in Table 2.

The interpretation of the results described in Section 3 requires their validation through an analysis of the soundscape; that is, the collection of sound sources present at a given location. As reported in Section 2.3, a single expert listened to all the recordings to quantify the amount of bio-, techno- and geo-phonies present at the recording site according to the categories reported in Table 1. The results of such a survey were mapped for the Parco Nord Milan area (Figure 17), and for Ticino River park (Figure 18).

For simplicity, we show two sound categories: number of birds (many) and traffic intensity (high). Figure 17 presents a clear separation of sites sitting close to the traffic noise sources (sites 1, 2, 3, 4, 5, 6 , 8) and those in the park’s interior (sites 17, 18, 19, 20, 22). The first group of sites contains the majority of recordings with high traffic noise intensity and a small fraction of them with many birds singing. The second group of sites displays the opposite trend: there was a low fraction of recordings with high traffic intensity and most recordings featured many birds singing. Such a result seems to suggest the connection between the presence of biophonic activity and TEM interconnections over a large spatial scale, whereas the presence of a high level of traffic noise seems to be responsible for a reduction in the extension of such network. These results are pretty much in agreement with those presented in Figure 15 and Figure 16.

Figure 18 illustrates the results for Ticino River park, revealing the distinct spatial distribution of biophonic and technophonic activity compared to Parco Nord Milan. Unlike the latter, where traffic noise constitutes the largest acoustic component in certain areas, the soundscape of TRP exhibits a more heterogeneous composition of auditory sources. Sites (7, 8, 9), in proximity to the A7 highway, showed a higher proportion of recordings influenced by intense traffic noise, whereas interior sites (1–6) exhibited a greater prevalence of bird vocalizations. However, in contrast to Parco Nord Milan, where biophonic activity was more consistently concentrated in the park’s core, the spatial distribution of biophony in Ticino River park is modulated by the diverse habitat types within the area. Notably, site 6, located near the oxbow, exhibited a pronounced biophonic presence despite its relative proximity to the highway, suggesting that localized environmental conditions exert a significant influence on the spatial dynamics of biophonic activity.

The analysis of Figure 18 quantifies the relationship between biophonic activity and the extent of the TEM interconnections. As observed in Parco Nord Milan, sites with higher bird vocalization rates tend to exhibit stronger TEM links, reinforcing the hypothesis that biophonic sources play a pivotal role in shaping acoustic connectivity. Conversely, areas dominated by traffic noise exhibited a reduction in intersite connections, suggesting that anthropogenic noise disrupts natural acoustic exchanges. These findings are in agreement with the patterns illustrated in Figure 15 and Figure 16, which depict intersite connectivity and the spatial distribution of TEM flows, further underscoring the contribution of biophonic activity to the structuring of acoustic networks in natural environments.

The use of a bivariate instead of a multivariate TEM represents a limitation of the present study, and some spurious interconnections could most likely be reduced using a multivariate approach [80,81]. Such extensions go beyond the scope of the present work. Indeed, a specific neuroscience study on people with schizophrenia suggests that a multivariate TEM outperforms a bivariate one under various signal-to-noise conditions [82]. However, it should be stressed that our approach was mainly devoted to establishing the presence of a network of interconnections among sites as a result of strong biophonic activities, rather than correctly predicting a causal relationship between a stimulus and response. Thus, this difference between our results and those obtained with a partial correction of spurious information flow, as in multivariate TEM, are probably not so important, although this remains to be determined.

TEM provides practical insights for park management and biodiversity conservation by identifying areas where acoustic connectivity is disrupted by anthropogenic noise. For example, in Parco Nord Milan, TEM highlighted zones near traffic sources with reduced bioacoustic connectivity, suggesting targeted actions like installing vegetation barriers or relocating pathways to minimize noise intrusion. In natural areas like Ticino River park, TEM can help prioritize the protection of ecologically connected zones that are critical for species communication, guiding effective conservation strategies.

In TRP, traditional indices like BI indicate high biophonic activity, but do not reveal how sounds propagate across sites. TEM identified a strong directional information flow during dawn, highlighting key areas for species interactions. Conversely, in Parco Nord Milan, TEM showed how traffic noise disrupts these connections, an insight that static indices alone cannot provide. This underscores TEM’s added value in detecting subtle perturbations within the acoustic environment, thus guiding more precise and effective management interventions.

## 5. Conclusions

Environment sounds are useful indicators of ecological features and degradation level within a local area [83,84]. However, the complexity of the overlapping sounds present in anthropized areas makes high-quality assessments of environmental sounds challenging. For this reason, a multidisciplinary approach, made of acoustic and ecoacoustic indicators, as well as ground-truthing, is becoming the common paradigm to obtain a proper understanding of these complex, multisource, overlapping components.

This approach becomes indispensable when, for instance, ecoacoustic indices are used outside the specific sound context for which they were devised. In this scenario, we applied the transfer entropy measure to add complementary information to the standard analysis. Indeed, TEM represents an improved tool to assess the nature of exchange information for many applications that require accurate estimations of both spatial and temporal connectivity among the empirically obtained time series.

In this paper, we used TEM, which refers either to Shannon (STE) or Rényi (RTE) transfer entropies, to extract spatio-temporal information on environmental sounds, which can be seen as complementary to the standard eco-acoustics approach. Specifically, we investigated the directional flow of acoustic information in urban (Parco Nord Milan) and natural (Ticino River park) green areas to better understand their ecological connectivity and the impact of anthropogenic noise. Shannon TE represents a very useful TEM since it obtains results that are quite independent of the chosen ecoacoustic index, whereas a weak dependence was observed in the case of Rényi TE. In this regard, STE can be considered to be a rather robust index of environment sound quality. The present work provides additional insights compared to the traditional approach based on eco-acoustic indices in both urban and wild environments, enabling a nuanced understanding of sound flow across varying anthropogenic and natural sound sources.

Biophonic soundscapes, recorded across multiple sites, tend to form dynamic networks of interconnections in space (see e.g., Figure 15a,b). A high level of information exchange can be observed by comparing the density of the interconnections or arrows between sites more exposed to antropized sounds with those that are more protected in the interior of the park. The spatial scale of interconnections is also influenced by disturbing non-natural sounds, which has the effect of reducing the length of these interconnections. From the TEM analysis, it becomes evident that there is a peak in connectivity at dawn, corresponding to heightened biophonic activity (see Figure 15c,d).

TEM extends ecoacoustic analytical research by incorporating a dynamic perspective of the soundscape connectivity, thereby enriching conventional methodologies. Such a directional analysis allows for a better understanding of how natural and anthropogenic sounds propagate through environments, providing valuable information for managing and preserving acoustic habitats. By identifying areas with possible disrupted bioacoustic connectivity, TEM can inform targeted conservation strategies and guide policy decisions aimed at mitigating noise pollution and preserving ecological integrity.

## Figures and Tables

**Table 1:** Labels assigned to the perceived sound categories in the aural survey activity and the corresponding attributes.

Category	Attribute
Birds singing	none , few , many
Bird species	none , ≤2 , >2
Singing activity (%)	0 , (0, 25] , (25, 50] , (50, 75] , (75, 100]
Traffic characteristics	none , continuous , intermittent
Traffic intensity	none , low , high
Other sound sources	none , presence

**Table 2:** Labels assigned to the perceived sound categories in the aural survey activity and their corresponding attributes.

**Parco Nord Milan Site**	**Many Birds**	**High Traffic**
1	0.04	1.00
2	0.06	1.00
3	0.19	1.00
4	0.10	1.00
5	0.21	0.99
6	0.24	0.66
8	0.46	1.00
10	0.51	0.00
11	0.79	0.00
12	0.76	0.19
13	0.61	0.20
17	0.84	0.00
18	0.74	0.01
19	0.67	0.29
20	0.76	0.00
22	0.69	0.00
**Ticino River park site**	**Many Birds**	**High Traffic**
1	0.33	0.03
2	0.50	0.00
3	0.31	0.01
4	0.31	0.00
6	0.58	0.00
7	0.24	1.00
8	0.48	0.97
9	0.48	1.00

## Data Availability

Data available upon request.

## Supplementary Materials

The following supporting information can be downloaded at: https://www.mdpi.com/article/10.3390/s25041046/s1.

## Appendix A. Dissimilarity vs. Kullback-Leibler Divergence

The concept of dissimilarity between distributions provides a simple way to estimate how ‘dissimilar’ (different) two distribution functions, say $P_{1}\left(x\right)$ and $P_{2}\left(x\right)$, are [50]. One can think of the subindices (1,2) as representing two different locations in space; the distributions are evaluated using acoustic sensors, separated by a distance of $R_{1,2}$. Their dissimilarity, $D_{1,2}$, is defined according to [50],

(A1) $$D_{1,2}=1-\frac{1}{A_{1}\;A_{2}}\int_{0}^{\infty }dx\;\;P_{1}\left(x\right)\;P_{2}\left(x\right),$$

with

(A2) $$A_{i}^{2}=\int_{0}^{\infty }dx\;\;P_{i}^{2}\left(x\right),\;i=1,2,$$

where the normalization factors $A_{i}$ ensure that $D_{i,i}=0$. The integral form used in Equation (A1) can formally be seen as the internal product of two vectors, $P_{1}\left(k\right)$ and $P_{2}\left(k\right)$, where k∈Z represents the coordinates in a high-dimensional space. Specifically, if we define the histograms $P_{i}\left(x\right)$ on a set of discrete coordinates, x=kb, where *b* is the bin size used to build the histograms, we may regard $P_{i}\left(k\right)$ as the *k*th component of the vector ${\bar{P}}_{i}$. Intuitively, the larger $D_{1,2}$ is, the greater the ‘distance’ between $P_{1}$ and $P_{2}$. We can loosely say that very different distribution functions are very distant in *x*-space.

In what follows, we present analytical results supportING the idea that dissimilarity is indeed closely related to the Kullback–Leibler divergence [61]. To do this, let us compare the KL-divergence and $D_{1,2}$ (Equation (A1)) corresponding to $P_{1}\left(x\right)$ and $P_{2}\left(x\right)$. This can be achieved analytically by considering the situation in which the distribution functions differ by a ‘small’ amount $\Delta P=P_{1}-P_{2}$, such that $\left|\Delta P\right|/P_{1,2}\ll 1$. In the case of the symmetrized KL divergence, we can write

(A3) $$\begin{array}{cc} D_{1,2}^{\mathrm{KL}} & =\frac{1}{2}\int_{0}^{\infty }du\;\left(P_{1}\log\frac{P_{1}}{P_{2}}+P_{2}\log\frac{P_{2}}{P_{1}}\right) \end{array}$$

(A4) $$\begin{array}{cc} \; & ≃\frac{1}{2}\int_{0}^{\infty }du\;\frac{P_{1}+P_{2}}{P_{1}P_{2}}{\left(\Delta P\right)}^{2}. \end{array}$$

The same approximation is applied to dissimilarity, $D_{1,2}$, Equation (A1), yielding

(A5) $$\begin{array}{cc} D_{1,2} & =1-\frac{1}{2}\frac{1}{A_{1}A_{2}}\int_{0}^{\infty }du\;\left[\left(P_{2}+\Delta P\right)P_{2}+P_{1}\left(P_{1}-\Delta P\right)\right] \end{array}$$

(A6) $$\begin{array}{cc} \; & ≃1-\frac{1}{2}\frac{1}{A_{1}A_{2}}\int_{0}^{\infty }du\;\left[P_{1}^{2}+P_{2}^{2}-{\left(\Delta P\right)}^{2}\right] \end{array}$$

(A7) $$\begin{array}{cc} \; & =1-\frac{1}{2}\frac{A_{1}^{2}+A_{2}^{2}}{A_{1}A_{2}}+\frac{1}{2}\frac{1}{A_{1}A_{2}}\int_{0}^{\infty }du\;{\left(\Delta P\right)}^{2}. \end{array}$$

In the case where both distributions differ slightly, one finds that $A_{1}≃A_{2}$, thus one has, $1-\left(A_{1}^{2}+A_{2}^{2}\right)/\left(2A_{1}A_{2}\right)=-{\left(A_{1}-A_{2}\right)}^{2}/\left(2A_{1}A_{2}\right)≲0$, yielding the result

(A8) $$D_{1,2}≃\frac{1}{2}\frac{1}{A_{1}A_{2}}\int_{0}^{\infty }du\;{\left(\Delta P\right)}^{2}.$$

If the factor $\left(P_{1}+P_{2}\right)/P_{1}P_{2}$ in the integrand of Equation (A4) behaves, after integration, as $∼\mathrm{const}/(A_{1}A_{2})$, then both $D_{1,2}^{\mathrm{KL}}$ and $D_{1,2}$ should display similar scaling dependencies with increasing distance, $R=R_{1,2}$, between the acoustic sensors. Indeed, we found that $D_{1,2}$ behaves as an approximate power law in the case of an urban park [50]

(A9) $$D_{1,2}\left(R\right)≃\;D_{0}\;{\left(R/R_{0}\right)}^{\beta },$$

and one can study the behavior of $D_{1,2}^{\mathrm{KL}}$ with *R* to check whether $D_{1,2}^{\mathrm{KL}}∼R^{\beta^{\prime }}$ also holds, eventually using a slightly different exponent $\beta^{\prime }\neq \beta$. Therefore, one can use two different methods, based on $D_{1,2}$ and $D_{1,2}^{\mathrm{KL}}$, to obtain more general conclusions about the spatial metric of the distributions.

## Appendix B. Shannon TE Matrix for Parco Nord Milan (Index H)

Here, we present examples of STE and DSTE for the Parco Nord Milan. The complete set of data can be found in the Supplementary Information file, and the raw data can be obtained upon request.

**Table A1:** Shannon TE matrix, STE (Equation (3)), for index H. Here, only values STE > 0.005 are retained, such that, in cases 0.010 < STE < 0.005, STE is rounded to 0.01. Each column represents the starting site for the STE. For instance, STE(1→2) = 0.14; STE(1→5) = 0.13; STE(2→1) = 0.02; and STE(5→1) = 0.03.

	1	2	3	4	5	6	8	10	11	12	13	17	18	19	20	22
1	0.00	0.02	0.01	0.14	0.03	0.10	0.01	0.01	0.03	0.09	0.01	0.01	0.02	0.01	0.14	0.01
2	0.14	0.00	0.02	0.01	0.07	0.02	0.01	0.01	0.03	0.04	0.02	0.07	0.02	0.02	0.01	0.01
3	0.01	0.23	0.00	0.02	0.05	0.02	0.01	0.02	0.01	0.02	0.02	0.01	0.01	0.01	0.02	0.02
4	0.01	0.03	0.01	0.00	0.01	0.01	0.01	0.02	0.01	0.02	0.10	0.03	0.14	0.02	0.01	0.01
5	0.13	0.01	0.01	0.01	0.00	0.03	0.01	0.01	0.02	0.04	0.03	0.14	0.04	0.01	0.01	0.01
6	0.02	0.05	0.03	0.01	0.01	0.00	0.01	0.02	0.03	0.04	0.04	0.03	0.13	0.01	0.01	0.02
8	0.01	0.04	0.01	0.02	0.01	0.04	0.00	0.01	0.02	0.01	0.02	0.01	0.01	0.02	0.01	0.02
10	0.01	0.01	0.01	0.01	0.01	0.01	0.01	0.00	0.01	0.02	0.02	0.01	0.01	0.01	0.01	0.02
11	0.05	0.06	0.08	0.01	0.03	0.04	0.01	0.02	0.00	0.01	0.03	0.02	0.05	0.01	0.01	0.02
12	0.02	0.03	0.01	0.03	0.02	0.01	0.02	0.01	0.02	0.00	0.05	0.03	0.08	0.01	0.01	0.02
13	0.02	0.01	0.02	0.01	0.03	0.01	0.01	0.01	0.01	0.03	0.00	0.02	0.01	0.02	0.01	0.01
17	0.02	0.01	0.01	0.10	0.01	0.06	0.01	0.02	0.05	0.04	0.02	0.00	0.04	0.02	0.39	0.02
18	0.04	0.02	0.01	0.02	0.01	0.02	0.02	0.01	0.01	0.04	0.02	0.02	0.00	0.01	0.02	0.02
19	0.04	0.01	0.01	0.01	0.01	0.01	0.01	0.01	0.01	0.02	0.02	0.01	0.01	0.00	0.03	0.02
20	0.03	0.01	0.01	0.01	0.01	0.02	0.01	0.02	0.01	0.05	0.13	0.01	0.09	0.02	0.00	0.02
22	0.01	0.03	0.03	0.01	0.01	0.01	0.01	0.01	0.03	0.01	0.01	0.05	0.01	0.03	0.07	0.00

**Table A2:** Difference Shannon matrix, DSTE (Equation (6)), for index H. After rounding the digits used, some values may differ in terms of the chosen cutoff, 0.01. Each column represents the starting site for the DSTE. Examples are as follows: DSTE(1→2) = 0.13; DSTE(1→5) = 0.09; DSTE(2→1) = 0.0; DSTE(5→1) = 0.0. Due to this rounding process, differences of up to 0.02 may arise.

	1	2	3	4	5	6	8	10	11	12	13	17	18	19	20	22
1	0.00	0.00	0.00	0.13	0.00	0.08	0.00	0.00	0.00	0.07	0.00	0.00	0.00	0.00	0.11	0.00
2	0.13	0.00	0.00	0.00	0.05	0.00	0.00	0.00	0.00	0.02	0.01	0.05	0.00	0.00	0.00	0.00
3	0.00	0.21	0.00	0.00	0.04	0.00	0.00	0.01	0.00	0.01	0.00	0.00	0.00	0.00	0.00	0.00
4	0.00	0.02	0.00	0.00	0.00	0.00	0.00	0.01	0.00	0.00	0.08	0.00	0.11	0.01	0.00	0.00
5	0.09	0.00	0.00	0.00	0.00	0.02	0.00	0.00	0.00	0.02	0.00	0.13	0.02	0.00	0.00	0.00
6	0.00	0.03	0.01	0.00	0.00	0.00	0.00	0.00	0.00	0.03	0.03	0.00	0.11	0.00	0.00	0.00
8	0.00	0.02	0.00	0.01	0.00	0.03	0.00	0.00	0.01	0.00	0.01	0.00	0.00	0.01	0.00	0.01
10	0.00	0.00	0.00	0.00	0.00	0.00	0.00	0.00	0.00	0.00	0.01	0.00	0.00	0.00	0.00	0.00
11	0.02	0.03	0.07	0.00	0.02	0.00	0.00	0.01	0.00	0.00	0.02	0.00	0.03	0.00	0.00	0.00
12	0.00	0.00	0.00	0.01	0.00	0.00	0.01	0.00	0.00	0.00	0.02	0.00	0.05	0.00	0.00	0.00
13	0.00	0.00	0.01	0.00	0.00	0.00	0.00	0.00	0.00	0.00	0.00	0.00	0.00	0.00	0.00	0.00
17	0.01	0.00	0.00	0.07	0.00	0.02	0.00	0.01	0.03	0.01	0.01	0.00	0.02	0.00	0.38	0.00
18	0.02	0.00	0.00	0.00	0.00	0.00	0.01	0.00	0.00	0.00	0.01	0.00	0.00	0.00	0.00	0.01
19	0.03	0.00	0.01	0.00	0.00	0.00	0.00	0.00	0.00	0.00	0.00	0.00	0.00	0.00	0.01	0.00
20	0.00	0.00	0.00	0.00	0.00	0.01	0.00	0.00	0.00	0.04	0.12	0.00	0.07	0.00	0.00	0.00
22	0.00	0.02	0.00	0.00	0.00	0.00	0.00	0.00	0.01	0.00	0.00	0.03	0.00	0.01	0.05	0.00
