# Supplementary material for: Application of Transfer Entropy Measure to Characterize Environmental Sounds in Urban and Wild Parks
Source: Sensors (Basel). 2025 Feb 10;25(4):1046. doi: 10.3390/s25041046 (PMC11859987; doi:10.3390/s25041046)
Supplement: Supplementary file 1 [file sensors-25-01046-s001.zip › sensors-3412567-supplementary.pdf]

Supplementary Information to the paper:

Application of Transfer Entropy Measure to Characterize Environmental Sounds in Urban and Wild Parks

Roberto Benocci, Giorgia Guagliumi, Andrea Potenza, Valentina Zaffaroni-Caorsi,
H. Eduardo Roman, and Giovanni Zambon

Content:

Parco Nord Milan (Shannon Transfer Entropy): H, BI

Table S1a: PNM STE INDEX H
Table S1b: PNM DSTE INDEX H <DSTE> = 0.026
Table S1c: PNM DSTE>0.026 INDEX H: Arrows

Table S2a: PNM STE INDEX BI
Table S2b: PNM DSTE INDEX BI <DSTE> = 0.011
Table S2c: PNM DSTE>0.011 INDEX BI: Arrows

Ticino River Park (Shannon Transfer Entropy) Dawn: H, BI

Table S3a: TRP STE INDEX H: DAWN
Table S3b: TRP DSTE INDEX H <DSTE> = 0.010
Table S3c: TRP DSTE>0.010 INDEX H: Arrows

Table S4a: TRP STE INDEX BI: DAWN
Table S4b: TRP DSTE INDEX BI <DSTE> = 0.009
Table S4c: TRP DSTE>0.009 INDEX BI: Arrows

Ticino River Park (Shannon Transfer Entropy) Day: H, BI

Table S5a: TRP STE INDEX H: DAY
Table S5b: TRP DSTE INDEX H <DSTE> = 0.006
Table S5c: TRP DSTE>0.006 INDEX H: Arrows

Table S6a: TRP STE INDEX BI: DAY
Table S6b: TRP DSTE INDEX BI <DSTE> = 0.003
Table S6c: TRP DSTE>0.003 INDEX BI: Arrows

Ticino River Park (Shannon Transfer Entropy) NIGHT: H, BI

Table S7a: TRP STE INDEX H: NIGHT
Table S7b: TRP DSTE INDEX H <DSTE> = 0.005
Table S7c: TRP DSTE>0.005 INDEX H: Arrows

Table S8a: TRP STE INDEX BI: NIGHT
Table S8b: TRP DSTE INDEX BI <DSTE> = 0.007
Table S8c: TRP DSTE>0.007 INDEX BI: Arrows

Parco Nord Milan (Renyi Transfer Entropy): H, BI q=0.1

Table S9a: PNM RTE INDEX H
Table S9b: PNM DRTE INDEX H   <DRTE> = 0.125
Table S9c: PNM DRTE>0.125   INDEX H: Arrows

Table S10a: PNM RTE INDEX BI
Table S10b: PNM DRTE INDEX BI <DRTE> = 0.109
Table S10c: PNM DRTE>0.109   INDEX BI: Arrows

Parco Nord Milan (Renyi Transfer Entropy): H, BI q=0.5

Table S11a: PNM RTE INDEX H
Table S11b: PNM DRTE INDEX H   <DRTE> = 0.057
Table S11c: PNM DRTE>0.057   INDEX H: Arrows

Table S12a: PNM RTE INDEX BI
Table S12b: PNM DRTE INDEX BI <DRTE> =0.053
Table S12c: PNM DRTE>0.053   INDEX BI: Arrows

Ticino River Park (Renyi Transfer Entropy) Dawn: H,BI q=0.1

Table S13a: TRP RTE INDEX H: DAWN
Table S13b: TRP DRTE INDEX H   <DRTE> =0.000
Table S13c: TRP DRTE>0.000   INDEX H: Arrows

Table S14a: TRP RTE INDEX BI: DAWN
Table S14b: TRP DRTE INDEX BI   <DRTE> =0.000
Table S14c: TRP DRTE>0.000   INDEX BI: Arrows

Ticino River Park (Renyi Transfer Entropy) Day: H,BI q=0.1

Table S15a: TRP RTE INDEX H: DAY
Table S15b: TRP DRTE INDEX H   <DRTE> =0.000
Table S15c: TRP DRTE>0.000   INDEX H: Arrows

Table S16a: TRP RTE INDEX BI: DAY
Table S16b: TRP DRTE INDEX BI   <DRTE> =0.000
Table S16c: TRP DRTE>0.000   INDEX BI: Arrows

Ticino River Park (Renyi Transfer Entropy) Night: H,BI q=0.1

Table S17a: TRP RTE INDEX H: NIGHT
Table S17b: TRP DRTE INDEX H   <DRTE> =0.000
Table S17c: TRP DRTE>0.000   INDEX H: Arrows

Table S18a: TRP RTE INDEX BI: NIGHT
Table S18b: TRP DRTE INDEX BI   <DRTE> =0.000
Table S18c: TRP DRTE>0.000   INDEX BI: Arrows

Ticino River Park (Renyi Transfer Entropy) Dawn: H,BI q=0.5

Table S19a: TRP RTE INDEX H: DAWN
Table S19b: TRP DRTE INDEX H <DRTE> =0.000
Table S19c: TRP DRTE>0.000 INDEX H: Arrows

Table S20a: TRP RTE INDEX BI: DAWN
Table S20b: TRP DRTE INDEX BI <DRTE> =0.000
Table S20c: TRP DRTE>0.000 INDEX BI: Arrows

Ticino River Park (Renyi Transfer Entropy) Day: H,BI q=0.5

Table S21a: TRP RTE INDEX H: DAY
Table S21b: TRP DRTE INDEX H <DRTE> =0.000
Table S21c: TRP DRTE>0.000 INDEX H: Arrows

Table S22a: TRP RTE INDEX BI: DAY
Table S22b: TRP DRTE INDEX BI <DRTE> =0.000
Table S22c: TRP DRTE>0.000 INDEX BI: Arrows

Ticino River Park (Renyi Transfer Entropy) Night: H,BI q=0.5

Table S23a: TRP RTE INDEX H: NIGHT
Table S23b: TRP DRTE INDEX H <DRTE> =0.000
Table S23c: TRP DRTE>0.000 INDEX H: Arrows

Table S24a: TRP RTE INDEX BI: NIGHT
Table S24b: TRP DRTE INDEX BI <DRTE> =0.000
Table S24c: TRP DRTE>0.000 INDEX BI: Arrows

Note on how to read the Tables:
Each column represents the starting site for the TEM or DTEM.

For instance, TEM = STE:
Table S1a: PNM STE INDEX H, the STE(1→2) = 0.14, STE(1→5) = 0.13,
And STE(2→1) = 0.02, STE(5→1) = 0.03.

The differences, DTEM, are reported as DSTE:
Table S1b: PNM DSTE INDEX H <DSTE> = 0.026, yielding,

DSTE(1→2) = STE(1→2) - STE(2→1) = 0.14 - 0.02 = 0.12 (up to rounding by 0.01)
DSTE(2→1)< 0 and assumed DSTE(2→1) = 0.

DSTE(1→5) = STE(1→5) - STE(5→1) = 0.13 - 0.03 = 0.10 (up to rounding by 0.01)
DSTE(5→1)< 0 and assumed DSTE(5→1) = 0.

All the Tables follow this convention.

Parco Nord Milan (Shannon Transfer Entropy): H

Table S1a: PNM STE INDEX H

	1	2	3	4	5	6	8	10	11	12	13	17	18	19	20	22
1	0.00	0.02	0.01	0.14	0.03	0.10	0.01	0.01	0.03	0.09	0.01	0.01	0.02	0.01	0.14	0.01
2	0.14	0.00	0.02	0.01	0.07	0.02	0.01	0.01	0.03	0.04	0.02	0.07	0.02	0.02	0.01	0.01
3	0.01	0.23	0.00	0.02	0.05	0.02	0.01	0.02	0.01	0.02	0.02	0.01	0.01	0.01	0.02	0.02
4	0.01	0.03	0.01	0.00	0.01	0.01	0.01	0.02	0.01	0.02	0.10	0.03	0.14	0.02	0.01	0.01
5	0.13	0.01	0.01	0.01	0.00	0.03	0.01	0.01	0.02	0.04	0.03	0.14	0.04	0.01	0.01	0.01
6	0.02	0.05	0.03	0.01	0.01	0.00	0.01	0.02	0.03	0.04	0.04	0.03	0.13	0.01	0.01	0.02
8	0.01	0.04	0.01	0.02	0.01	0.04	0.00	0.01	0.02	0.01	0.02	0.01	0.01	0.02	0.01	0.02
10	0.01	0.01	0.01	0.01	0.01	0.01	0.01	0.00	0.01	0.02	0.02	0.01	0.01	0.01	0.01	0.02
11	0.05	0.06	0.08	0.01	0.03	0.04	0.01	0.02	0.00	0.01	0.03	0.02	0.05	0.01	0.01	0.02
12	0.02	0.03	0.01	0.03	0.02	0.01	0.02	0.01	0.02	0.00	0.05	0.03	0.08	0.01	0.01	0.02
13	0.02	0.01	0.02	0.01	0.03	0.01	0.01	0.01	0.01	0.03	0.00	0.02	0.01	0.02	0.01	0.01
17	0.02	0.01	0.01	0.10	0.01	0.06	0.01	0.02	0.05	0.04	0.02	0.00	0.04	0.02	0.39	0.02
18	0.04	0.02	0.01	0.02	0.01	0.02	0.02	0.01	0.01	0.04	0.02	0.02	0.00	0.01	0.02	0.02
19	0.04	0.01	0.01	0.01	0.01	0.01	0.01	0.01	0.01	0.02	0.02	0.01	0.01	0.00	0.03	0.02
20	0.03	0.01	0.01	0.01	0.01	0.02	0.01	0.02	0.01	0.05	0.13	0.01	0.09	0.02	0.00	0.02
22	0.01	0.03	0.03	0.01	0.01	0.01	0.01	0.01	0.03	0.01	0.01	0.05	0.01	0.03	0.07	0.00

Table S1b: PNM DSTE INDEX H <DSTE> = 0.026

	1	2	3	4	5	6	8	10	11	12	13	17	18	19	20	22
1	0.00	0.00	0.00	0.13	0.00	0.08	0.00	0.00	0.00	0.07	0.00	0.00	0.00	0.00	0.11	0.00
2	0.13	0.00	0.00	0.00	0.05	0.00	0.00	0.00	0.00	0.02	0.01	0.05	0.00	0.00	0.00	0.00
3	0.00	0.21	0.00	0.00	0.04	0.00	0.00	0.01	0.00	0.01	0.00	0.00	0.00	0.00	0.00	0.00
4	0.00	0.02	0.00	0.00	0.00	0.00	0.00	0.01	0.00	0.00	0.08	0.00	0.11	0.01	0.00	0.00
5	0.09	0.00	0.00	0.00	0.00	0.02	0.00	0.00	0.00	0.02	0.00	0.13	0.02	0.00	0.00	0.00
6	0.00	0.03	0.01	0.00	0.00	0.00	0.00	0.00	0.00	0.03	0.03	0.00	0.11	0.00	0.00	0.00
8	0.00	0.02	0.00	0.01	0.00	0.03	0.00	0.00	0.01	0.00	0.01	0.00	0.00	0.01	0.00	0.01
10	0.00	0.00	0.00	0.00	0.00	0.00	0.00	0.00	0.00	0.00	0.01	0.00	0.00	0.00	0.00	0.00
11	0.02	0.03	0.07	0.00	0.02	0.00	0.00	0.01	0.00	0.00	0.02	0.00	0.03	0.00	0.00	0.00
12	0.00	0.00	0.00	0.01	0.00	0.00	0.01	0.00	0.00	0.00	0.02	0.00	0.05	0.00	0.00	0.00
13	0.00	0.00	0.01	0.00	0.00	0.00	0.00	0.00	0.00	0.00	0.00	0.00	0.00	0.00	0.00	0.00
17	0.01	0.00	0.00	0.07	0.00	0.02	0.00	0.01	0.03	0.01	0.01	0.00	0.02	0.00	0.38	0.00
18	0.02	0.00	0.00	0.00	0.00	0.00	0.01	0.00	0.00	0.00	0.01	0.00	0.00	0.00	0.00	0.01
19	0.03	0.00	0.01	0.00	0.00	0.00	0.00	0.00	0.00	0.00	0.00	0.00	0.00	0.00	0.01	0.00
20	0.00	0.00	0.00	0.00	0.00	0.01	0.00	0.00	0.00	0.04	0.12	0.00	0.07	0.00	0.00	0.00
22	0.00	0.02	0.00	0.00	0.00	0.00	0.00	0.00	0.01	0.00	0.00	0.03	0.00	0.01	0.05	0.00

Table S1c: PNM DSTE>0.026 INDEX H: Arrows

	1	2	3	4	5	6	8	10	11	12	13	17	18	19	20	22
1	0.00	0.00	0.00	0.13	0.00	0.08	0.00	0.00	0.00	0.07	0.00	0.00	0.00	0.00	0.11	0.00
2	0.13	0.00	0.00	0.00	0.05	0.00	0.00	0.00	0.00	0.00	0.00	0.05	0.00	0.00	0.00	0.00
3	0.00	0.21	0.00	0.00	0.04	0.00	0.00	0.00	0.00	0.00	0.00	0.00	0.00	0.00	0.00	0.00
4	0.00	0.00	0.00	0.00	0.00	0.00	0.00	0.00	0.00	0.00	0.08	0.00	0.11	0.00	0.00	0.00
5	0.09	0.00	0.00	0.00	0.00	0.00	0.00	0.00	0.00	0.00	0.00	0.13	0.00	0.00	0.00	0.00
6	0.00	0.03	0.00	0.00	0.00	0.00	0.00	0.00	0.00	0.03	0.03	0.00	0.11	0.00	0.00	0.00
8	0.00	0.00	0.00	0.00	0.00	0.03	0.00	0.00	0.00	0.00	0.00	0.00	0.00	0.00	0.00	0.00
10	0.00	0.00	0.00	0.00	0.00	0.00	0.00	0.00	0.00	0.00	0.00	0.00	0.00	0.00	0.00	0.00
11	0.00	0.03	0.07	0.00	0.00	0.00	0.00	0.00	0.00	0.00	0.00	0.00	0.03	0.00	0.00	0.00
12	0.00	0.00	0.00	0.00	0.00	0.00	0.00	0.00	0.00	0.00	0.00	0.00	0.05	0.00	0.00	0.00
13	0.00	0.00	0.00	0.00	0.00	0.00	0.00	0.00	0.00	0.00	0.00	0.00	0.00	0.00	0.00	0.00
17	0.00	0.00	0.00	0.07	0.00	0.00	0.00	0.00	0.03	0.00	0.00	0.00	0.00	0.00	0.38	0.00
18	0.00	0.00	0.00	0.00	0.00	0.00	0.00	0.00	0.00	0.00	0.00	0.00	0.00	0.00	0.00	0.00
19	0.00	0.00	0.00	0.00	0.00	0.00	0.00	0.00	0.00	0.00	0.00	0.00	0.00	0.00	0.00	0.00
20	0.00	0.00	0.00	0.00	0.00	0.00	0.00	0.00	0.00	0.04	0.12	0.00	0.07	0.00	0.00	0.00
22	0.00	0.00	0.00	0.00	0.00	0.00	0.00	0.00	0.00	0.00	0.00	0.03	0.00	0.00	0.05	0.00

Parco Nord Milan (Shannon Transfer Entropy): BI

Table S2a: PNM STE INDEX BI

	1	2	3	4	5	6	8	10	11	12	13	17	18	19	20	22
1	0.00	0.01	0.01	0.02	0.03	0.01	0.02	0.02	0.03	0.01	0.02	0.04	0.04	0.01	0.02	0.01
2	0.03	0.00	0.05	0.02	0.02	0.04	0.02	0.01	0.02	0.05	0.02	0.01	0.03	0.02	0.03	0.02
3	0.01	0.14	0.00	0.03	0.03	0.01	0.01	0.05	0.02	0.02	0.01	0.08	0.04	0.02	0.01	0.03
4	0.01	0.02	0.06	0.00	0.02	0.03	0.04	0.02	0.01	0.02	0.01	0.01	0.02	0.01	0.04	0.01
5	0.02	0.02	0.01	0.07	0.00	0.02	0.03	0.02	0.01	0.02	0.01	0.02	0.01	0.02	0.01	0.02
6	0.01	0.01	0.01	0.01	0.01	0.00	0.01	0.02	0.02	0.02	0.01	0.02	0.03	0.01	0.03	0.01
8	0.01	0.01	0.05	0.02	0.02	0.01	0.00	0.04	0.01	0.01	0.01	0.02	0.01	0.03	0.03	0.02
10	0.04	0.02	0.03	0.02	0.02	0.03	0.03	0.00	0.01	0.03	0.01	0.04	0.03	0.02	0.04	0.01
11	0.02	0.02	0.01	0.01	0.01	0.02	0.01	0.01	0.00	0.01	0.03	0.03	0.02	0.01	0.01	0.01
12	0.01	0.01	0.01	0.02	0.03	0.01	0.03	0.02	0.01	0.00	0.01	0.02	0.01	0.01	0.01	0.01
13	0.03	0.02	0.01	0.02	0.01	0.01	0.04	0.01	0.02	0.02	0.00	0.01	0.03	0.06	0.03	0.02
17	0.01	0.02	0.04	0.03	0.02	0.03	0.04	0.02	0.01	0.01	0.02	0.00	0.01	0.01	0.02	0.05
18	0.04	0.01	0.02	0.01	0.02	0.01	0.01	0.02	0.01	0.01	0.01	0.02	0.00	0.02	0.04	0.04
19	0.01	0.01	0.01	0.02	0.03	0.01	0.02	0.01	0.01	0.01	0.01	0.01	0.04	0.00	0.02	0.05
20	0.01	0.05	0.02	0.01	0.01	0.02	0.02	0.04	0.01	0.01	0.01	0.02	0.04	0.03	0.00	0.02
22	0.04	0.01	0.02	0.01	0.03	0.01	0.01	0.02	0.02	0.01	0.01	0.04	0.01	0.03	0.02	0.00

Table S2b: PNM DSTE INDEX BI <DSTE> = 0.011

	1	2	3	4	5	6	8	10	11	12	13	17	18	19	20	22
1	0.00	0.00	0.00	0.00	0.02	0.00	0.01	0.00	0.01	0.00	0.00	0.02	0.00	0.00	0.00	0.00
2	0.01	0.00	0.00	0.00	0.00	0.03	0.01	0.00	0.00	0.04	0.00	0.00	0.02	0.01	0.00	0.00
3	0.00	0.09	0.00	0.00	0.01	0.00	0.00	0.02	0.01	0.00	0.00	0.03	0.02	0.00	0.00	0.01
4	0.00	0.00	0.02	0.00	0.00	0.01	0.02	0.00	0.00	0.00	0.00	0.00	0.01	0.00	0.03	0.00
5	0.00	0.00	0.00	0.06	0.00	0.00	0.01	0.00	0.00	0.00	0.00	0.00	0.00	0.00	0.00	0.00
6	0.00	0.00	0.00	0.00	0.00	0.00	0.00	0.00	0.00	0.01	0.01	0.00	0.01	0.00	0.01	0.00
8	0.00	0.00	0.03	0.00	0.00	0.00	0.00	0.02	0.00	0.00	0.00	0.00	0.00	0.01	0.01	0.01
10	0.02	0.01	0.00	0.01	0.00	0.00	0.00	0.00	0.00	0.01	0.00	0.01	0.02	0.02	0.00	0.00
11	0.00	0.00	0.00	0.00	0.00	0.00	0.00	0.01	0.00	0.00	0.01	0.03	0.01	0.00	0.00	0.00
12	0.00	0.00	0.00	0.00	0.01	0.00	0.02	0.00	0.00	0.00	0.00	0.01	0.00	0.00	0.00	0.00
13	0.01	0.00	0.00	0.00	0.00	0.00	0.02	0.00	0.00	0.00	0.00	0.00	0.02	0.05	0.02	0.01
17	0.00	0.00	0.00	0.02	0.00	0.01	0.02	0.00	0.00	0.00	0.01	0.00	0.00	0.00	0.00	0.01
18	0.00	0.00	0.00	0.00	0.01	0.00	0.00	0.00	0.00	0.00	0.00	0.01	0.00	0.00	0.00	0.02
19	0.00	0.00	0.00	0.00	0.01	0.00	0.00	0.00	0.00	0.00	0.00	0.00	0.03	0.00	0.00	0.02
20	0.00	0.02	0.01	0.00	0.00	0.00	0.00	0.00	0.00	0.00	0.00	0.01	0.00	0.01	0.00	0.00
22	0.02	0.00	0.00	0.00	0.01	0.00	0.00	0.01	0.01	0.00	0.00	0.00	0.00	0.00	0.00	0.00

Table S2c: PNM DSTE>0.011 INDEX BI: Arrows

	1	2	3	4	5	6	8	10	11	12	13	17	18	19	20	22
1	0.00	0.00	0.00	0.00	0.02	0.00	0.01	0.00	0.00	0.00	0.00	0.02	0.00	0.00	0.00	0.00
2	0.01	0.00	0.00	0.00	0.00	0.03	0.00	0.00	0.00	0.04	0.00	0.00	0.02	0.00	0.00	0.00
3	0.00	0.09	0.00	0.00	0.01	0.00	0.00	0.02	0.00	0.00	0.00	0.03	0.02	0.00	0.00	0.00
4	0.00	0.00	0.02	0.00	0.00	0.01	0.02	0.00	0.00	0.00	0.00	0.00	0.00	0.00	0.03	0.00
5	0.00	0.00	0.00	0.06	0.00	0.00	0.00	0.00	0.00	0.00	0.00	0.00	0.00	0.00	0.00	0.00
6	0.00	0.00	0.00	0.00	0.00	0.00	0.00	0.00	0.00	0.00	0.00	0.00	0.01	0.00	0.01	0.00
8	0.00	0.00	0.03	0.00	0.00	0.00	0.00	0.02	0.00	0.00	0.00	0.00	0.00	0.00	0.01	0.01
10	0.02	0.00	0.00	0.00	0.00	0.00	0.00	0.00	0.00	0.00	0.00	0.01	0.02	0.02	0.00	0.00
11	0.00	0.00	0.00	0.00	0.00	0.00	0.00	0.00	0.00	0.00	0.00	0.03	0.00	0.00	0.00	0.00
12	0.00	0.00	0.00	0.00	0.00	0.00	0.02	0.00	0.00	0.00	0.00	0.00	0.00	0.00	0.00	0.00
13	0.01	0.00	0.00	0.00	0.00	0.00	0.02	0.00	0.00	0.00	0.00	0.00	0.02	0.05	0.02	0.01
17	0.00	0.00	0.00	0.02	0.00	0.00	0.02	0.00	0.00	0.00	0.01	0.00	0.00	0.00	0.00	0.00
18	0.00	0.00	0.00	0.00	0.00	0.00	0.00	0.00	0.00	0.00	0.00	0.00	0.00	0.00	0.00	0.02
19	0.00	0.00	0.00	0.00	0.01	0.00	0.00	0.00	0.00	0.00	0.00	0.00	0.03	0.00	0.00	0.02
20	0.00	0.02	0.00	0.00	0.00	0.00	0.00	0.00	0.00	0.00	0.00	0.00	0.00	0.00	0.00	0.00
22	0.02	0.00	0.00	0.00	0.01	0.00	0.00	0.00	0.00	0.00	0.00	0.00	0.00	0.00	0.00	0.00

Ticino River Park (Shannon Transfer Entropy) Dawn: H, BI

Table S3a: TRP STE INDEX H: DAWN

	1	2	3	4	6	7	8	9
1	0.00	0.04	0.01	0.02	0.03	0.02	0.03	0.01
2	0.03	0.00	0.03	0.02	0.03	0.02	0.02	0.01
3	0.02	0.04	0.00	0.03	0.04	0.04	0.01	0.01
4	0.01	0.01	0.01	0.00	0.04	0.02	0.01	0.01
6	0.03	0.02	0.03	0.02	0.00	0.03	0.02	0.01
7	0.02	0.02	0.01	0.03	0.02	0.00	0.01	0.02
8	0.05	0.01	0.02	0.02	0.02	0.05	0.00	0.01
9	0.02	0.01	0.01	0.02	0.02	0.02	0.01	0.00

Table S3b: TRP DSTE INDEX H <DSTE> = 0.010

	1	2	3	4	6	7	8	9
1	0.00	0.00	0.00	0.01	0.00	0.00	0.00	0.00
2	0.00	0.00	0.00	0.01	0.01	0.00	0.00	0.00
3	0.02	0.01	0.00	0.01	0.01	0.03	0.00	0.00
4	0.00	0.00	0.00	0.00	0.02	0.00	0.00	0.00
6	0.00	0.00	0.00	0.00	0.00	0.01	0.00	0.00
7	0.00	0.00	0.00	0.01	0.00	0.00	0.00	0.00
8	0.02	0.00	0.00	0.01	0.00	0.04	0.00	0.00
9	0.01	0.00	0.00	0.01	0.01	0.00	0.00	0.00

Table S3c: TRP DSTE>0.010 INDEX H: Arrows

	1	2	3	4	6	7	8	9
1	0.00	0.00	0.00	0.00	0.00	0.00	0.00	0.00
2	0.00	0.00	0.00	0.00	0.00	0.00	0.00	0.00
3	0.02	0.01	0.00	0.01	0.00	0.03	0.00	0.00
4	0.00	0.00	0.00	0.00	0.02	0.00	0.00	0.00
6	0.00	0.00	0.00	0.00	0.00	0.01	0.00	0.00
7	0.00	0.00	0.00	0.00	0.00	0.00	0.00	0.00
8	0.02	0.00	0.00	0.00	0.00	0.04	0.00	0.00
9	0.00	0.00	0.00	0.00	0.00	0.00	0.00	0.00

Table S4a: TRP STE INDEX BI: DAWN

	1	2	3	4	6	7	8	9
1	0.00	0.04	0.03	0.05	0.05	0.03	0.04	0.01
2	0.05	0.00	0.04	0.04	0.03	0.04	0.03	0.01
3	0.03	0.02	0.00	0.04	0.02	0.04	0.04	0.02
4	0.05	0.03	0.04	0.00	0.02	0.05	0.04	0.02
6	0.05	0.05	0.02	0.05	0.00	0.04	0.05	0.01
7	0.03	0.03	0.02	0.03	0.02	0.00	0.04	0.02
8	0.04	0.04	0.02	0.03	0.02	0.03	0.00	0.01
9	0.01	0.01	0.01	0.02	0.01	0.01	0.01	0.00

Table S4b: TRP DSTE INDEX BI <DSTE> = 0.009

	1	2	3	4	6	7	8	9
1	0.00	0.00	0.01	0.00	0.00	0.00	0.00	0.00
2	0.01	0.00	0.02	0.01	0.00	0.01	0.00	0.00
3	0.00	0.00	0.00	0.00	0.00	0.02	0.01	0.00
4	0.00	0.00	0.00	0.00	0.00	0.02	0.01	0.00
6	0.01	0.02	0.00	0.03	0.00	0.02	0.02	0.00
7	0.00	0.00	0.00	0.00	0.00	0.00	0.01	0.00
8	0.00	0.00	0.00	0.00	0.00	0.00	0.00	0.00
9	0.00	0.00	0.00	0.00	0.00	0.00	0.00	0.00

Table S4c: TRP DSTE>0.009 INDEX BI: Arrows

	1	2	3	4	6	7	8	9
1	0.00	0.00	0.00	0.00	0.00	0.00	0.00	0.00
2	0.01	0.00	0.02	0.01	0.00	0.01	0.00	0.00
3	0.00	0.00	0.00	0.00	0.00	0.02	0.01	0.00
4	0.00	0.00	0.00	0.00	0.00	0.02	0.01	0.00
6	0.00	0.02	0.00	0.03	0.00	0.02	0.02	0.00
7	0.00	0.00	0.00	0.00	0.00	0.00	0.01	0.00
8	0.00	0.00	0.00	0.00	0.00	0.00	0.00	0.00
9	0.00	0.00	0.00	0.00	0.00	0.00	0.00	0.00

Ticino River Park (Shannon Transfer Entropy) Day: H, BI

Table S5a: TRP STE INDEX H: DAY

	1	2	3	4	6	7	8	9
1	0.00	0.01	0.02	0.01	0.01	0.01	0.01	0.01
2	0.02	0.00	0.02	0.03	0.02	0.03	0.02	0.01
3	0.03	0.01	0.00	0.02	0.02	0.02	0.02	0.01
4	0.02	0.03	0.02	0.00	0.02	0.01	0.01	0.01
6	0.02	0.03	0.03	0.03	0.00	0.02	0.02	0.01
7	0.02	0.03	0.02	0.02	0.02	0.00	0.01	0.02
8	0.01	0.01	0.02	0.01	0.01	0.01	0.00	0.01
9	0.01	0.01	0.01	0.01	0.01	0.02	0.02	0.00

Table S5b: TRP DSTE INDEX H <DSTE> = 0.006

	1	2	3	4	6	7	8	9
1	0.00	0.00	0.00	0.00	0.00	0.00	0.00	0.00
2	0.01	0.00	0.01	0.00	0.00	0.00	0.01	0.00
3	0.02	0.00	0.00	0.00	0.00	0.00	0.00	0.00
4	0.00	0.00	0.00	0.00	0.00	0.00	0.00	0.00
6	0.01	0.01	0.01	0.01	0.00	0.01	0.01	0.00
7	0.01	0.00	0.00	0.01	0.00	0.00	0.01	0.00
8	0.00	0.00	0.00	0.00	0.00	0.00	0.00	0.00
9	0.00	0.00	0.00	0.00	0.01	0.00	0.01	0.00

Table S5c: TRP DSTE>0.006 INDEX H: Arrows

	1	2	3	4	6	7	8	9
1	0.00	0.00	0.00	0.00	0.00	0.00	0.00	0.00
2	0.01	0.00	0.01	0.00	0.00	0.00	0.00	0.00
3	0.02	0.00	0.00	0.00	0.00	0.00	0.00	0.00
4	0.00	0.00	0.00	0.00	0.00	0.00	0.00	0.00
6	0.00	0.01	0.00	0.01	0.00	0.01	0.01	0.00
7	0.01	0.00	0.00	0.01	0.00	0.00	0.01	0.00
8	0.00	0.00	0.00	0.00	0.00	0.00	0.00	0.00
9	0.00	0.00	0.00	0.00	0.01	0.00	0.01	0.00

Table S6a: TRP STE INDEX BI: DAY

	1	2	3	4	6	7	8	9
1	0.00	0.01	0.02	0.01	0.01	0.02	0.02	0.00
2	0.01	0.00	0.02	0.01	0.03	0.02	0.02	0.00
3	0.02	0.02	0.00	0.01	0.02	0.02	0.03	0.01
4	0.01	0.02	0.01	0.00	0.01	0.02	0.01	0.01
6	0.01	0.01	0.02	0.00	0.00	0.03	0.02	0.00
7	0.01	0.01	0.02	0.01	0.01	0.00	0.02	0.01
8	0.01	0.02	0.02	0.01	0.02	0.02	0.00	0.00
9	0.01	0.00	0.00	0.01	0.01	0.01	0.00	0.00

Table S6b: TRP DSTE INDEX BI <DSTE> = 0.003

	1	2	3	4	6	7	8	9
1	0.00	0.00	0.00	0.00	0.00	0.00	0.01	0.00
2	0.00	0.00	0.00	0.00	0.01	0.00	0.00	0.00
3	0.00	0.00	0.00	0.00	0.00	0.00	0.01	0.00
4	0.00	0.00	0.00	0.00	0.00	0.00	0.00	0.00
6	0.00	0.00	0.00	0.00	0.00	0.01	0.00	0.00
7	0.00	0.00	0.00	0.00	0.00	0.00	0.00	0.00
8	0.00	0.00	0.00	0.00	0.00	0.00	0.00	0.00
9	0.00	0.00	0.00	0.00	0.00	0.00	0.00	0.00

Table S6c: TRP DSTE>0.003 INDEX BI: Arrows

	1	2	3	4	6	7	8	9
1	0.00	0.00	0.00	0.00	0.00	0.00	0.01	0.00
2	0.00	0.00	0.00	0.00	0.01	0.00	0.00	0.00
3	0.00	0.00	0.00	0.00	0.00	0.00	0.01	0.00
4	0.00	0.00	0.00	0.00	0.00	0.00	0.00	0.00
6	0.00	0.00	0.00	0.00	0.00	0.01	0.00	0.00
7	0.00	0.00	0.00	0.00	0.00	0.00	0.00	0.00
8	0.00	0.00	0.00	0.00	0.00	0.00	0.00	0.00
9	0.00	0.00	0.00	0.00	0.00	0.00	0.00	0.00

Ticino River Park (Shannon Transfer Entropy) NIGHT: H, BI

Table S7a: TRP STE INDEX H: NIGHT

	1	2	3	4	6	7	8	9
1	0.00	0.02	0.01	0.01	0.02	0.01	0.02	0.01
2	0.01	0.00	0.02	0.01	0.01	0.01	0.01	0.01
3	0.01	0.02	0.00	0.03	0.02	0.02	0.02	0.01
4	0.01	0.02	0.01	0.00	0.01	0.02	0.02	0.01
6	0.02	0.01	0.02	0.02	0.00	0.02	0.02	0.01
7	0.01	0.01	0.02	0.02	0.01	0.00	0.01	0.01
8	0.01	0.01	0.01	0.01	0.01	0.01	0.00	0.01
9	0.02	0.02	0.02	0.02	0.02	0.02	0.02	0.00

Table S7b: TRP DSTE INDEX H <DSTE> = 0.005

	1	2	3	4	6	7	8	9
1	0.00	0.00	0.00	0.00	0.00	0.00	0.01	0.00
2	0.00	0.00	0.00	0.00	0.00	0.00	0.01	0.00
3	0.00	0.00	0.00	0.01	0.01	0.00	0.01	0.00
4	0.00	0.01	0.00	0.00	0.00	0.00	0.01	0.00
6	0.00	0.00	0.00	0.00	0.00	0.00	0.01	0.00
7	0.00	0.00	0.00	0.00	0.00	0.00	0.00	0.00
8	0.00	0.00	0.00	0.00	0.00	0.00	0.00	0.00
9	0.01	0.01	0.01	0.01	0.01	0.01	0.01	0.00

Table S7c: TRP DSTE>0.005 INDEX H: Arrows

	1	2	3	4	6	7	8	9
1	0.00	0.00	0.00	0.00	0.00	0.00	0.01	0.00
2	0.00	0.00	0.00	0.00	0.00	0.00	0.01	0.00
3	0.00	0.00	0.00	0.01	0.01	0.00	0.01	0.00
4	0.00	0.01	0.00	0.00	0.00	0.00	0.01	0.00
6	0.00	0.00	0.00	0.00	0.00	0.00	0.01	0.00
7	0.00	0.00	0.00	0.00	0.00	0.00	0.00	0.00
8	0.00	0.00	0.00	0.00	0.00	0.00	0.00	0.00
9	0.01	0.01	0.01	0.01	0.01	0.01	0.01	0.00

Table S8a: TRP STE INDEX BI: NIGHT

	1	2	3	4	6	7	8	9
1	0.00	0.03	0.02	0.02	0.02	0.02	0.03	0.02
2	0.03	0.00	0.03	0.02	0.04	0.02	0.03	0.02
3	0.01	0.02	0.00	0.01	0.02	0.03	0.01	0.01
4	0.03	0.03	0.01	0.00	0.02	0.01	0.01	0.02
6	0.03	0.03	0.03	0.01	0.00	0.02	0.04	0.01
7	0.01	0.02	0.02	0.01	0.01	0.00	0.01	0.01
8	0.02	0.02	0.02	0.02	0.02	0.03	0.00	0.01
9	0.02	0.01	0.00	0.02	0.01	0.01	0.01	0.00

Table S8b: TRP DSTE INDEX BI <DSTE> = 0.007

	1	2	3	4	6	7	8	9
1	0.00	0.00	0.01	0.00	0.00	0.01	0.01	0.00
2	0.00	0.00	0.01	0.00	0.01	0.00	0.00	0.01
3	0.00	0.00	0.00	0.00	0.00	0.00	0.00	0.01
4	0.01	0.01	0.00	0.00	0.01	0.00	0.00	0.00
6	0.01	0.00	0.01	0.00	0.00	0.01	0.02	0.01
7	0.00	0.00	0.00	0.00	0.00	0.00	0.00	0.00
8	0.00	0.00	0.01	0.00	0.00	0.02	0.00	0.01
9	0.00	0.00	0.00	0.00	0.00	0.00	0.00	0.00

Table S8c: TRP DSTE>0.007 INDEX BI: Arrows

	1	2	3	4	6	7	8	9
1	0.00	0.00	0.00	0.00	0.00	0.01	0.01	0.00
2	0.00	0.00	0.00	0.00	0.01	0.00	0.00	0.01
3	0.00	0.00	0.00	0.00	0.00	0.00	0.00	0.01
4	0.01	0.01	0.00	0.00	0.01	0.00	0.00	0.00
6	0.00	0.00	0.00	0.00	0.00	0.01	0.02	0.00
7	0.00	0.00	0.00	0.00	0.00	0.00	0.00	0.00
8	0.00	0.00	0.00	0.00	0.00	0.02	0.00	0.01
9	0.00	0.00	0.00	0.00	0.00	0.00	0.00	0.00

Parco Nord Milan (Renyi Transfer Entropy): H q=0.1

Table S9a: PNM RTE INDEX H

	1	2	3	4	5	6	8	10	11	12	13	17	18	19	20	22
1	0.00	0.48	0.21	0.01	0.45	0.12	0.30	0.19	-0.01	0.27	0.13	0.42	0.20	0.31	0.25	0.15
2	0.19	0.00	0.38	0.23	0.11	0.31	0.19	0.31	0.24	0.30	0.38	0.30	0.24	0.19	0.37	0.43
3	0.21	0.37	0.00	0.37	0.15	0.43	0.24	0.20	0.21	0.31	0.31	0.37	0.43	0.02	0.15	0.20
4	0.21	0.48	0.19	0.00	0.12	0.32	0.11	0.37	0.15	0.29	0.02	0.03	0.25	0.38	0.37	0.30
5	0.30	0.36	0.21	0.23	0.00	0.25	0.02	0.11	0.01	0.39	0.23	0.12	0.32	0.24	0.24	0.30
6	0.31	0.55	0.50	0.31	0.01	0.00	0.25	0.43	0.24	0.19	0.10	0.12	0.13	0.24	0.37	0.37
8	0.38	0.35	0.19	-0.01	0.30	0.16	0.00	0.12	0.43	0.19	0.24	0.42	0.24	0.31	0.23	0.23
10	0.27	0.44	0.38	0.15	0.11	0.31	0.11	0.00	0.30	0.45	0.11	0.30	-0.10	-0.10	0.08	0.48
11	0.39	0.36	0.39	0.15	0.25	0.00	0.31	0.02	0.00	0.20	0.16	0.43	0.30	0.02	0.20	0.31
12	0.28	0.37	0.30	0.19	0.20	0.20	0.23	0.27	0.12	0.00	0.09	0.00	0.10	0.24	0.24	0.37
13	0.31	0.25	0.27	0.15	0.23	0.20	0.12	0.12	0.17	0.39	0.00	0.37	0.20	0.30	0.30	0.30
17	0.38	0.44	0.43	0.15	0.24	0.23	0.30	0.19	0.09	0.19	0.38	0.00	0.31	0.09	0.60	0.16
18	0.50	0.07	0.38	0.10	0.20	0.26	0.23	0.15	0.19	0.39	0.21	0.20	0.00	0.12	0.01	0.23
19	0.38	0.44	0.34	0.24	0.37	0.24	0.30	0.30	0.15	0.20	0.30	0.30	0.01	0.00	0.37	0.36
20	0.43	0.47	0.34	0.37	0.12	0.24	0.24	0.31	0.10	0.31	0.16	0.24	0.11	0.16	0.00	0.20
22	0.21	0.45	0.39	0.30	0.15	0.37	0.12	0.48	0.19	0.43	0.15	0.16	0.12	0.36	0.10	0.00

Table S9b: PNM DRTE INDEX H <DRTE> = 0.125

	1	2	3	4	5	6	8	10	11	12	13	17	18	19	20	22
1	0.00	0.28	0.00	0.00	0.15	0.00	0.00	0.00	0.00	0.00	0.00	0.04	0.00	0.00	0.00	0.00
2	0.00	0.00	0.02	0.00	0.00	0.00	0.00	0.00	0.00	0.00	0.12	0.00	0.17	0.00	0.00	0.00
3	0.00	0.00	0.00	0.18	0.00	0.00	0.05	0.00	0.00	0.00	0.04	0.00	0.05	0.00	0.00	0.00
4	0.20	0.24	0.00	0.00	0.00	0.01	0.12	0.22	0.00	0.10	0.00	0.00	0.14	0.14	0.00	0.00
5	0.00	0.25	0.06	0.12	0.00	0.23	0.00	0.00	0.00	0.19	0.00	0.00	0.11	0.00	0.12	0.14
6	0.19	0.24	0.07	0.00	0.00	0.00	0.10	0.12	0.23	0.00	0.00	0.00	0.00	0.00	0.13	0.00
8	0.08	0.15	0.00	0.00	0.28	0.00	0.00	0.00	0.13	0.00	0.13	0.12	0.01	0.01	0.00	0.12
10	0.08	0.14	0.19	0.00	0.00	0.00	0.00	0.00	0.28	0.18	0.00	0.11	0.00	0.00	0.00	0.00
11	0.40	0.12	0.19	0.00	0.24	0.00	0.00	0.00	0.00	0.08	0.00	0.33	0.11	0.00	0.10	0.12
12	0.01	0.06	0.00	0.00	0.00	0.01	0.04	0.00	0.00	0.00	0.00	0.00	0.00	0.05	0.00	0.00
13	0.18	0.00	0.00	0.13	0.00	0.11	0.00	0.00	0.00	0.29	0.00	0.00	0.00	0.00	0.14	0.14
17	0.00	0.15	0.06	0.12	0.12	0.11	0.00	0.00	0.00	0.18	0.01	0.00	0.11	0.00	0.36	0.00
18	0.30	0.00	0.00	0.00	0.00	0.12	0.00	0.26	0.00	0.29	0.00	0.00	0.00	0.11	0.00	0.11
19	0.08	0.26	0.32	0.00	0.13	0.00	0.00	0.40	0.13	0.00	0.00	0.21	0.00	0.00	0.21	0.00
20	0.18	0.10	0.19	0.00	0.00	0.00	0.00	0.23	0.00	0.07	0.00	0.00	0.10	0.00	0.00	0.10
22	0.06	0.02	0.19	0.00	0.00	0.00	0.00	0.00	0.00	0.06	0.00	0.00	0.00	0.00	0.00	0.00

Table S9c: PNM DRTE>0.125 INDEX H: Arrows

	1	2	3	4	5	6	8	10	11	12	13	17	18	19	20	22
1	0.00	0.28	0.00	0.00	0.15	0.00	0.00	0.00	0.00	0.00	0.00	0.00	0.00	0.00	0.00	0.00
2	0.00	0.00	0.00	0.00	0.00	0.00	0.00	0.00	0.00	0.00	0.00	0.00	0.17	0.00	0.00	0.00
3	0.00	0.00	0.00	0.18	0.00	0.00	0.00	0.00	0.00	0.00	0.00	0.00	0.00	0.00	0.00	0.00
4	0.20	0.24	0.00	0.00	0.00	0.00	0.00	0.22	0.00	0.00	0.00	0.00	0.14	0.14	0.00	0.00
5	0.00	0.25	0.00	0.00	0.00	0.23	0.00	0.00	0.00	0.19	0.00	0.00	0.00	0.00	0.00	0.14
6	0.19	0.24	0.00	0.00	0.00	0.00	0.00	0.00	0.23	0.00	0.00	0.00	0.00	0.00	0.13	0.00
8	0.00	0.15	0.00	0.00	0.28	0.00	0.00	0.00	0.13	0.00	0.13	0.00	0.00	0.00	0.00	0.00
10	0.00	0.14	0.19	0.00	0.00	0.00	0.00	0.00	0.28	0.18	0.00	0.00	0.00	0.00	0.00	0.00
11	0.40	0.12	0.19	0.00	0.24	0.00	0.00	0.00	0.00	0.00	0.00	0.33	0.00	0.00	0.00	0.00
12	0.00	0.00	0.00	0.00	0.00	0.00	0.00	0.00	0.00	0.00	0.00	0.00	0.00	0.00	0.00	0.00
13	0.18	0.00	0.00	0.13	0.00	0.00	0.00	0.00	0.00	0.29	0.00	0.00	0.00	0.00	0.14	0.14
17	0.00	0.15	0.00	0.00	0.00	0.00	0.00	0.00	0.00	0.18	0.00	0.00	0.00	0.00	0.36	0.00
18	0.30	0.00	0.00	0.00	0.00	0.00	0.00	0.26	0.00	0.29	0.00	0.00	0.00	0.00	0.00	0.00
19	0.00	0.26	0.32	0.00	0.13	0.00	0.00	0.40	0.13	0.00	0.00	0.21	0.00	0.00	0.21	0.00
20	0.18	0.00	0.19	0.00	0.00	0.00	0.00	0.23	0.00	0.00	0.00	0.00	0.00	0.00	0.00	0.00
22	0.00	0.00	0.19	0.00	0.00	0.00	0.00	0.00	0.00	0.00	0.00	0.00	0.00	0.00	0.00	0.00

Parco Nord Milan (Renyi Transfer Entropy): BI q=0.1

Table S10a: PNM RTE INDEX BI

	1	2	3	4	5	6	8	10	11	12	13	17	18	19	20	22
1	0.00	0.20	0.30	0.40	0.28	0.09	0.45	0.39	0.29	0.21	0.32	0.67	0.39	0.21	0.43	0.40
2	0.38	0.00	0.27	0.29	0.19	-0.03	-0.03	0.32	0.50	0.40	0.26	0.26	0.29	0.50	0.35	0.50
3	0.19	0.09	0.00	0.28	0.29	0.40	0.38	0.39	0.31	0.50	0.11	0.50	0.39	0.45	0.21	0.18
4	0.50	0.31	0.37	0.00	0.29	0.19	0.40	0.31	0.09	0.40	0.43	0.37	0.10	0.39	0.20	0.21
5	0.27	0.10	0.38	0.39	0.00	0.31	0.20	0.39	0.21	0.09	0.37	0.26	0.38	0.39	0.34	0.28
6	0.34	0.02	0.49	0.19	0.43	0.00	0.30	0.20	0.50	0.39	0.30	0.45	0.29	0.35	0.18	0.34
8	0.44	0.02	0.27	0.29	0.20	0.31	0.00	0.35	0.30	0.21	0.37	0.44	0.43	0.09	0.45	0.44
10	0.37	0.11	0.27	0.30	0.50	0.31	0.56	0.00	0.34	0.19	0.15	0.44	0.28	0.21	0.26	0.38
11	0.38	0.31	0.30	0.21	0.21	0.39	0.43	0.21	0.00	0.34	0.31	0.37	0.31	0.35	0.34	0.39
12	0.08	0.21	0.27	0.39	0.19	0.28	0.08	0.30	0.21	0.00	0.31	0.25	0.43	0.31	0.34	0.34
13	0.38	0.26	-0.02	0.50	0.31	0.09	0.08	0.21	0.38	0.40	0.00	0.44	0.19	0.26	-0.00	0.51
17	0.38	0.09	0.31	0.10	0.19	0.39	0.28	0.28	0.31	0.43	0.30	0.00	0.43	0.19	0.38	0.28
18	0.37	0.10	0.27	0.40	0.39	0.39	0.30	0.29	0.43	0.31	0.08	0.48	0.00	0.10	0.27	0.28
19	0.21	0.31	0.25	0.39	0.28	0.21	0.19	0.09	0.34	0.31	0.15	0.25	0.20	0.00	0.35	0.18
20	0.19	0.25	0.19	0.32	0.35	0.28	0.45	0.45	0.21	0.34	0.00	0.45	0.36	0.35	0.00	0.21
22	0.18	0.19	0.27	0.34	0.38	0.21	0.08	0.39	0.38	0.34	0.31	0.67	0.29	0.30	-0.03	0.00

Table S10b: PNM DRTE INDEX BI <DRTE> = 0.109

	1	2	3	4	5	6	8	10	11	12	13	17	18	19	20	22
1	0.00	0.00	0.11	0.00	0.01	0.00	0.01	0.01	0.00	0.13	0.00	0.29	0.02	0.01	0.24	0.22
2	0.18	0.00	0.17	0.00	0.10	0.00	0.00	0.21	0.19	0.20	0.00	0.17	0.19	0.19	0.10	0.31
3	0.00	0.00	0.00	0.00	0.00	0.00	0.11	0.12	0.01	0.23	0.14	0.19	0.12	0.20	0.01	0.00
4	0.10	0.02	0.10	0.00	0.00	0.00	0.11	0.01	0.00	0.01	0.00	0.27	0.00	0.00	0.00	0.00
5	0.00	0.00	0.09	0.10	0.00	0.00	0.00	0.00	0.00	0.00	0.06	0.07	0.00	0.11	0.00	0.00
6	0.25	0.05	0.10	0.00	0.12	0.00	0.00	0.00	0.11	0.11	0.21	0.05	0.00	0.13	0.00	0.13
8	0.00	0.05	0.00	0.00	0.00	0.00	0.00	0.00	0.00	0.13	0.29	0.17	0.12	0.00	0.01	0.35
10	0.00	0.00	0.00	0.00	0.12	0.10	0.21	0.00	0.13	0.00	0.00	0.16	0.00	0.12	0.00	0.00
11	0.09	0.00	0.00	0.13	0.01	0.00	0.13	0.00	0.00	0.13	0.00	0.05	0.00	0.00	0.14	0.00
12	0.00	0.00	0.00	0.00	0.10	0.00	0.00	0.11	0.00	0.00	0.00	0.00	0.12	0.00	0.00	0.00
13	0.06	0.00	0.00	0.07	0.00	0.00	0.00	0.06	0.07	0.09	0.00	0.14	0.10	0.10	0.00	0.20
17	0.00	0.00	0.00	0.00	0.00	0.00	0.00	0.00	0.00	0.18	0.00	0.00	0.00	0.00	0.00	0.00
18	0.00	0.00	0.00	0.30	0.00	0.10	0.00	0.01	0.12	0.00	0.00	0.05	0.00	0.00	0.00	0.00
19	0.00	0.00	0.00	0.00	0.00	0.00	0.09	0.00	0.00	0.00	0.00	0.06	0.10	0.00	0.00	0.00
20	0.00	0.00	0.00	0.12	0.00	0.10	0.00	0.19	0.00	0.00	0.01	0.06	0.10	0.00	0.00	0.24
22	0.00	0.00	0.09	0.13	0.10	0.00	0.00	0.01	0.00	0.00	0.00	0.39	0.01	0.11	0.00	0.00

Table S10c: PNM DRTE>0.109 INDEX BI: Arrows

	1	2	3	4	5	6	8	10	11	12	13	17	18	19	20	22
1	0.00	0.00	0.11	0.00	0.00	0.00	0.00	0.00	0.00	0.13	0.00	0.29	0.00	0.00	0.24	0.22
2	0.18	0.00	0.17	0.00	0.00	0.00	0.00	0.21	0.19	0.20	0.00	0.17	0.19	0.19	0.00	0.31
3	0.00	0.00	0.00	0.00	0.00	0.00	0.11	0.12	0.00	0.23	0.14	0.19	0.12	0.20	0.00	0.00
4	0.00	0.00	0.00	0.00	0.00	0.00	0.00	0.00	0.00	0.00	0.00	0.27	0.00	0.00	0.00	0.00
5	0.00	0.00	0.00	0.00	0.00	0.00	0.00	0.00	0.00	0.00	0.00	0.00	0.00	0.11	0.00	0.00
6	0.25	0.00	0.00	0.00	0.12	0.00	0.00	0.00	0.11	0.00	0.21	0.00	0.00	0.13	0.00	0.13
8	0.00	0.00	0.00	0.00	0.00	0.00	0.00	0.00	0.00	0.13	0.29	0.17	0.12	0.00	0.00	0.35
10	0.00	0.00	0.00	0.00	0.12	0.00	0.21	0.00	0.13	0.00	0.00	0.16	0.00	0.12	0.00	0.00
11	0.00	0.00	0.00	0.13	0.00	0.00	0.13	0.00	0.00	0.13	0.00	0.00	0.00	0.00	0.14	0.00
12	0.00	0.00	0.00	0.00	0.00	0.00	0.00	0.11	0.00	0.00	0.00	0.00	0.12	0.00	0.00	0.00
13	0.00	0.00	0.00	0.00	0.00	0.00	0.00	0.00	0.00	0.00	0.00	0.14	0.00	0.00	0.00	0.20
17	0.00	0.00	0.00	0.00	0.00	0.00	0.00	0.00	0.00	0.18	0.00	0.00	0.00	0.00	0.00	0.00
18	0.00	0.00	0.00	0.30	0.00	0.00	0.00	0.00	0.12	0.00	0.00	0.00	0.00	0.00	0.00	0.00
19	0.00	0.00	0.00	0.00	0.00	0.00	0.00	0.00	0.00	0.00	0.00	0.00	0.00	0.00	0.00	0.00
20	0.00	0.00	0.00	0.12	0.00	0.00	0.00	0.19	0.00	0.00	0.00	0.00	0.00	0.00	0.00	0.24
22	0.00	0.00	0.00	0.13	0.00	0.00	0.00	0.00	0.00	0.00	0.00	0.39	0.00	0.11	0.00	0.00

Parco Nord Milan (Renyi Transfer Entropy): H q=0.5

Table S11a: PNM RTE INDEX H

	1	2	3	4	5	6	8	10	11	12	13	17	18	19	20	22
1	0.00	0.21	0.11	0.06	0.23	0.09	0.13	0.07	-0.02	0.11	0.08	0.17	0.10	0.13	0.18	0.10
2	0.12	0.00	0.16	0.11	0.06	0.14	0.08	0.13	0.12	0.14	0.19	0.13	0.12	0.06	0.17	0.19
3	0.11	0.25	0.00	0.17	0.09	0.20	0.11	0.09	0.10	0.14	0.13	0.16	0.19	0.03	0.09	0.08
4	0.11	0.22	0.07	0.00	0.06	0.14	0.06	0.16	0.09	0.12	0.07	0.05	0.18	0.18	0.16	0.13
5	0.19	0.15	0.11	0.11	0.00	0.14	0.03	0.05	0.02	0.16	0.11	0.12	0.17	0.11	0.11	0.15
6	0.14	0.23	0.23	0.13	0.03	0.00	0.09	0.20	0.12	0.06	0.04	0.08	0.13	0.11	0.16	0.17
8	0.15	0.12	0.07	-0.01	0.16	0.05	0.00	0.06	0.20	0.07	0.13	0.18	0.12	0.14	0.11	0.10
10	0.09	0.17	0.15	0.03	0.05	0.13	0.06	0.00	0.16	0.17	0.05	0.11	-0.02	-0.02	0.01	0.20
11	0.18	0.17	0.19	0.09	0.15	0.01	0.13	0.03	0.00	0.10	0.06	0.19	0.14	0.04	0.09	0.14
12	0.11	0.17	0.13	0.08	0.09	0.08	0.10	0.12	0.06	0.00	0.04	0.01	0.06	0.12	0.10	0.17
13	0.14	0.09	0.09	0.09	0.11	0.09	0.06	0.06	0.06	0.16	0.00	0.17	0.09	0.12	0.15	0.15
17	0.15	0.17	0.19	0.12	0.11	0.12	0.13	0.08	0.05	0.07	0.19	0.00	0.15	0.02	0.56	0.05
18	0.23	0.01	0.15	0.05	0.10	0.15	0.11	0.10	0.08	0.17	0.10	0.09	0.00	0.06	0.03	0.10
19	0.17	0.17	0.17	0.11	0.18	0.12	0.13	0.16	0.09	0.08	0.12	0.12	0.01	0.00	0.16	0.15
20	0.20	0.20	0.17	0.16	0.06	0.11	0.11	0.14	0.04	0.15	0.15	0.11	0.07	0.05	0.00	0.10
22	0.11	0.18	0.17	0.13	0.10	0.17	0.06	0.21	0.08	0.19	0.10	0.07	0.06	0.15	0.07	0.00

Table S11b: PNM DRTE INDEX H <DRTE> = 0.057

	1	2	3	4	5	6	8	10	11	12	13	17	18	19	20	22
1	0.00	0.08	0.00	0.00	0.04	0.00	0.00	0.00	0.00	0.00	0.00	0.02	0.00	0.00	0.00	0.00
2	0.00	0.00	0.00	0.00	0.00	0.00	0.00	0.00	0.00	0.00	0.10	0.00	0.11	0.00	0.00	0.01
3	0.00	0.10	0.00	0.10	0.00	0.00	0.04	0.00	0.00	0.01	0.05	0.00	0.04	0.00	0.00	0.00
4	0.05	0.10	0.00	0.00	0.00	0.01	0.07	0.13	0.00	0.05	0.00	0.00	0.14	0.07	0.00	0.00
5	0.00	0.09	0.01	0.05	0.00	0.11	0.00	0.00	0.00	0.08	0.00	0.00	0.07	0.00	0.05	0.05
6	0.06	0.09	0.03	0.00	0.00	0.00	0.04	0.06	0.11	0.00	0.00	0.00	0.00	0.00	0.05	0.00
8	0.02	0.05	0.00	0.00	0.13	0.00	0.00	0.00	0.07	0.00	0.07	0.05	0.01	0.01	0.00	0.04
10	0.02	0.03	0.06	0.00	0.00	0.00	0.00	0.00	0.13	0.05	0.00	0.03	0.00	0.00	0.00	0.00
11	0.20	0.05	0.09	0.00	0.13	0.00	0.00	0.00	0.00	0.04	0.00	0.14	0.06	0.00	0.05	0.06
12	0.00	0.03	0.00	0.00	0.00	0.02	0.03	0.00	0.00	0.00	0.00	0.00	0.00	0.04	0.00	0.00
13	0.06	0.00	0.00	0.03	0.00	0.05	0.00	0.01	0.00	0.12	0.00	0.00	0.00	0.00	0.00	0.06
17	0.00	0.03	0.03	0.07	0.00	0.04	0.00	0.00	0.00	0.06	0.02	0.00	0.05	0.00	0.46	0.00
18	0.13	0.00	0.00	0.00	0.00	0.02	0.00	0.12	0.00	0.10	0.01	0.00	0.00	0.05	0.00	0.04
19	0.03	0.10	0.14	0.00	0.06	0.00	0.00	0.18	0.06	0.00	0.00	0.09	0.00	0.00	0.11	0.00
20	0.02	0.04	0.08	0.01	0.00	0.00	0.01	0.13	0.00	0.05	0.00	0.00	0.04	0.00	0.00	0.03
22	0.01	0.00	0.09	0.00	0.00	0.00	0.00	0.00	0.00	0.02	0.00	0.01	0.00	0.00	0.00	0.00

Table S11c: PNM DRTE>0.057 INDEX H: Arrows

	1	2	3	4	5	6	8	10	11	12	13	17	18	19	20	22
1	0.00	0.08	0.00	0.00	0.00	0.00	0.00	0.00	0.00	0.00	0.00	0.00	0.00	0.00	0.00	0.00
2	0.00	0.00	0.00	0.00	0.00	0.00	0.00	0.00	0.00	0.00	0.10	0.00	0.11	0.00	0.00	0.00
3	0.00	0.10	0.00	0.10	0.00	0.00	0.00	0.00	0.00	0.00	0.00	0.00	0.00	0.00	0.00	0.00
4	0.00	0.10	0.00	0.00	0.00	0.00	0.07	0.13	0.00	0.00	0.00	0.00	0.14	0.07	0.00	0.00
5	0.00	0.09	0.00	0.00	0.00	0.11	0.00	0.00	0.00	0.08	0.00	0.00	0.07	0.00	0.00	0.00
6	0.00	0.09	0.00	0.00	0.00	0.00	0.00	0.06	0.11	0.00	0.00	0.00	0.00	0.00	0.00	0.00
8	0.00	0.00	0.00	0.00	0.13	0.00	0.00	0.00	0.07	0.00	0.07	0.00	0.00	0.00	0.00	0.00
10	0.00	0.00	0.06	0.00	0.00	0.00	0.00	0.00	0.13	0.00	0.00	0.00	0.00	0.00	0.00	0.00
11	0.20	0.00	0.09	0.00	0.13	0.00	0.00	0.00	0.00	0.00	0.00	0.14	0.06	0.00	0.00	0.06
12	0.00	0.00	0.00	0.00	0.00	0.00	0.00	0.00	0.00	0.00	0.00	0.00	0.00	0.00	0.00	0.00
13	0.00	0.00	0.00	0.00	0.00	0.00	0.00	0.00	0.00	0.12	0.00	0.00	0.00	0.00	0.00	0.00
17	0.00	0.00	0.00	0.07	0.00	0.00	0.00	0.00	0.00	0.06	0.00	0.00	0.00	0.00	0.46	0.00
18	0.13	0.00	0.00	0.00	0.00	0.00	0.00	0.12	0.00	0.10	0.00	0.00	0.00	0.00	0.00	0.00
19	0.00	0.10	0.14	0.00	0.06	0.00	0.00	0.18	0.00	0.00	0.00	0.09	0.00	0.00	0.11	0.00
20	0.00	0.00	0.08	0.00	0.00	0.00	0.00	0.13	0.00	0.00	0.00	0.00	0.00	0.00	0.00	0.00
22	0.00	0.00	0.09	0.00	0.00	0.00	0.00	0.00	0.00	0.00	0.00	0.00	0.00	0.00	0.00	0.00

Parco Nord Milan (Renyi Transfer Entropy): BI q=0.5

Table S12a: PNM RTE INDEX BI

	1	2	3	4	5	6	8	10	11	12	13	17	18	19	20	22
1	0.00	0.09	0.13	0.18	0.11	0.05	0.19	0.15	0.11	0.11	0.16	0.29	0.17	0.11	0.19	0.17
2	0.15	0.00	0.08	0.13	0.07	-0.01	-0.01	0.16	0.21	0.20	0.10	0.08	0.12	0.22	0.11	0.21
3	0.07	0.08	0.00	0.10	0.11	0.18	0.15	0.16	0.13	0.21	0.06	0.20	0.17	0.17	0.09	0.04
4	0.21	0.15	0.15	0.00	0.12	0.07	0.19	0.13	0.04	0.17	0.19	0.16	0.02	0.15	0.10	0.11
5	0.09	0.04	0.14	0.18	0.00	0.13	0.09	0.16	0.10	0.02	0.17	0.08	0.15	0.16	0.17	0.10
6	0.17	0.03	0.21	0.08	0.19	0.00	0.13	0.09	0.21	0.16	0.16	0.17	0.12	0.17	0.04	0.17
8	0.16	0.03	0.09	0.12	0.08	0.13	0.00	0.12	0.12	0.11	0.17	0.17	0.19	0.04	0.19	0.21
10	0.14	0.05	0.08	0.12	0.22	0.13	0.24	0.00	0.16	0.08	0.10	0.17	0.10	0.11	0.08	0.15
11	0.14	0.14	0.13	0.11	0.11	0.15	0.19	0.11	0.00	0.16	0.15	0.16	0.14	0.17	0.17	0.15
12	0.05	0.10	0.09	0.16	0.07	0.10	0.05	0.12	0.10	0.00	0.13	0.08	0.19	0.13	0.17	0.17
13	0.16	0.10	-0.04	0.22	0.13	0.05	0.01	0.10	0.15	0.17	0.00	0.17	0.07	0.08	-0.01	0.23
17	0.15	0.03	0.07	0.04	0.07	0.17	0.11	0.10	0.14	0.18	0.13	0.00	0.19	0.08	0.15	0.10
18	0.14	0.04	0.08	0.18	0.16	0.15	0.13	0.12	0.18	0.13	0.02	0.21	0.00	0.06	0.09	0.11
19	0.11	0.13	0.05	0.16	0.10	0.11	0.07	0.05	0.16	0.13	0.04	0.09	0.10	0.00	0.12	0.06
20	0.07	0.08	0.08	0.15	0.17	0.10	0.17	0.17	0.10	0.16	-0.00	0.18	0.15	0.12	0.00	0.11
22	0.05	0.07	0.08	0.17	0.16	0.11	0.02	0.16	0.15	0.16	0.13	0.30	0.12	0.15	-0.01	0.00

Table S12b: PNM DRTE INDEX BI <DRTE> =0.053

	1	2	3	4	5	6	8	10	11	12	13	17	18	19	20	22
1	0.00	0.00	0.06	0.00	0.02	0.00	0.03	0.01	0.00	0.06	0.01	0.15	0.03	0.01	0.12	0.12
2	0.05	0.00	0.00	0.00	0.03	0.00	0.00	0.11	0.07	0.10	0.00	0.05	0.08	0.08	0.03	0.14
3	0.00	0.00	0.00	0.00	0.00	0.00	0.06	0.09	0.00	0.12	0.10	0.12	0.09	0.12	0.01	0.00
4	0.02	0.02	0.05	0.00	0.00	0.00	0.07	0.01	0.00	0.02	0.00	0.12	0.00	0.00	0.00	0.00
5	0.00	0.00	0.03	0.05	0.00	0.00	0.01	0.00	0.00	0.00	0.04	0.01	0.00	0.06	0.00	0.00
6	0.12	0.04	0.03	0.01	0.06	0.00	0.00	0.00	0.05	0.06	0.11	0.00	0.00	0.06	0.00	0.06
8	0.00	0.05	0.00	0.00	0.00	0.00	0.00	0.00	0.00	0.06	0.16	0.06	0.06	0.00	0.02	0.19
10	0.00	0.00	0.00	0.00	0.07	0.04	0.12	0.00	0.04	0.00	0.00	0.07	0.00	0.06	0.00	0.00
11	0.03	0.00	0.00	0.07	0.02	0.00	0.07	0.00	0.00	0.06	0.01	0.02	0.00	0.01	0.07	0.00
12	0.00	0.00	0.00	0.00	0.05	0.00	0.00	0.05	0.00	0.00	0.00	0.00	0.07	0.00	0.00	0.01
13	0.00	0.00	0.00	0.02	0.00	0.00	0.00	0.01	0.00	0.04	0.00	0.04	0.06	0.05	0.00	0.09
17	0.00	0.00	0.00	0.00	0.00	0.00	0.00	0.00	0.00	0.10	0.00	0.00	0.00	0.00	0.00	0.00
18	0.00	0.00	0.00	0.16	0.01	0.03	0.00	0.02	0.04	0.00	0.00	0.02	0.00	0.00	0.00	0.00
19	0.00	0.00	0.00	0.00	0.00	0.00	0.03	0.00	0.00	0.00	0.00	0.01	0.05	0.00	0.01	0.00
20	0.00	0.00	0.00	0.06	0.00	0.06	0.00	0.09	0.00	0.00	0.01	0.03	0.06	0.00	0.00	0.12
22	0.00	0.00	0.04	0.06	0.06	0.00	0.00	0.01	0.00	0.00	0.00	0.19	0.01	0.09	0.00	0.00

Table S12c: PNM DRTE>0.053   INDEX BI: Arrows

	1	2	3	4	5	6	8	10	11	12	13	17	18	19	20	22
1	0.00	0.00	0.06	0.00	0.00	0.00	0.00	0.00	0.00	0.06	0.00	0.15	0.00	0.00	0.12	0.12
2	0.00	0.00	0.00	0.00	0.00	0.00	0.00	0.11	0.07	0.10	0.00	0.05	0.08	0.08	0.00	0.14
3	0.00	0.00	0.00	0.00	0.00	0.00	0.06	0.09	0.00	0.12	0.10	0.12	0.09	0.12	0.00	0.00
4	0.00	0.00	0.00	0.00	0.00	0.00	0.07	0.00	0.00	0.00	0.00	0.12	0.00	0.00	0.00	0.00
5	0.00	0.00	0.00	0.00	0.00	0.00	0.00	0.00	0.00	0.00	0.00	0.00	0.00	0.06	0.00	0.00
6	0.12	0.00	0.00	0.00	0.06	0.00	0.00	0.00	0.00	0.06	0.11	0.00	0.00	0.06	0.00	0.06
8	0.00	0.00	0.00	0.00	0.00	0.00	0.00	0.00	0.00	0.06	0.16	0.06	0.06	0.00	0.00	0.19
10	0.00	0.00	0.00	0.00	0.07	0.00	0.12	0.00	0.00	0.00	0.00	0.07	0.00	0.06	0.00	0.00
11	0.00	0.00	0.00	0.07	0.00	0.00	0.07	0.00	0.00	0.06	0.00	0.00	0.00	0.00	0.07	0.00
12	0.00	0.00	0.00	0.00	0.05	0.00	0.00	0.00	0.00	0.00	0.00	0.00	0.07	0.00	0.00	0.00
13	0.00	0.00	0.00	0.00	0.00	0.00	0.00	0.00	0.00	0.00	0.00	0.00	0.06	0.00	0.00	0.09
17	0.00	0.00	0.00	0.00	0.00	0.00	0.00	0.00	0.00	0.10	0.00	0.00	0.00	0.00	0.00	0.00
18	0.00	0.00	0.00	0.16	0.00	0.00	0.00	0.00	0.00	0.00	0.00	0.00	0.00	0.00	0.00	0.00
19	0.00	0.00	0.00	0.00	0.00	0.00	0.00	0.00	0.00	0.00	0.00	0.00	0.00	0.00	0.00	0.00
20	0.00	0.00	0.00	0.06	0.00	0.06	0.00	0.09	0.00	0.00	0.00	0.00	0.06	0.00	0.00	0.12
22	0.00	0.00	0.00	0.06	0.06	0.00	0.00	0.00	0.00	0.00	0.00	0.19	0.00	0.09	0.00	0.00

Ticino River Park (Renyi Transfer Entropy) Dawn: H q=0.1

Table S13a: TRP RTE INDEX H: DAWN

	1	2	3	4	6	7	8	9
1	0.00	0.10	0.19	0.11	0.19	0.18	0.10	0.32
2	0.11	0.00	0.10	0.11	0.20	0.28	0.11	0.32
3	0.10	0.10	0.00	0.11	0.20	0.39	0.11	0.21
4	0.19	0.11	0.10	0.00	0.19	0.28	0.21	0.21
6	0.19	0.10	0.19	0.10	0.00	0.19	0.21	0.32
7	0.19	0.20	0.29	0.11	0.19	0.00	0.11	0.36
8	0.01	0.10	0.10	0.02	0.19	0.01	0.00	0.45
9	0.21	0.22	0.21	0.20	0.21	0.18	0.18	0.00

Table S13b: TRP DRTE INDEX H <DRTE> =0.069

	1	2	3	4	6	7	8	9
1	0.00	0.00	0.09	0.00	0.00	0.00	0.09	0.11
2	0.01	0.00	0.00	0.00	0.09	0.09	0.00	0.10
3	0.00	0.00	0.00	0.01	0.01	0.10	0.01	0.00
4	0.08	0.00	0.00	0.00	0.09	0.17	0.18	0.00
6	0.00	0.00	0.00	0.00	0.00	0.00	0.02	0.11
7	0.00	0.00	0.00	0.00	0.00	0.00	0.09	0.18
8	0.00	0.00	0.00	0.00	0.00	0.00	0.00	0.28
9	0.00	0.00	0.00	0.00	0.00	0.00	0.00	0.00

Table S13c: TRP DRTE>0.069 INDEX H: Arrows

	1	2	3	4	6	7	8	9
1	0.00	0.00	0.09	0.00	0.00	0.00	0.09	0.11
2	0.00	0.00	0.00	0.00	0.09	0.09	0.00	0.10
3	0.00	0.00	0.00	0.00	0.00	0.10	0.00	0.00
4	0.08	0.00	0.00	0.00	0.09	0.17	0.18	0.00
6	0.00	0.00	0.00	0.00	0.00	0.00	0.00	0.11
7	0.00	0.00	0.00	0.00	0.00	0.00	0.09	0.18
8	0.00	0.00	0.00	0.00	0.00	0.00	0.00	0.28
9	0.00	0.00	0.00	0.00	0.00	0.00	0.00	0.00

Ticino River Park (Renyi Transfer Entropy) Dawn: BI q=0.1

Table S14a: TRP RTE INDEX BI: DAWN

	1	2	3	4	6	7	8	9
1	0.00	0.13	0.19	0.30	0.18	0.39	0.31	0.29
2	0.22	0.00	0.20	0.30	0.19	0.31	0.20	0.29
3	0.40	0.20	0.00	0.31	0.12	0.33	0.22	0.39
4	0.41	0.50	0.31	0.00	0.29	0.41	0.28	0.50
6	0.28	0.31	0.21	0.31	0.00	0.37	0.30	0.31
7	0.19	0.29	0.22	0.30	0.27	0.00	0.36	0.39
8	0.21	0.20	0.22	0.20	0.21	0.45	0.00	0.31
9	0.29	0.28	0.29	0.28	0.10	0.28	0.20	0.00

Table S14b: TRP DRTE INDEX BI <DRTE> =0.093

	1	2	3	4	6	7	8	9
1	0.00	0.00	0.00	0.00	0.00	0.20	0.09	0.00
2	0.09	0.00	0.00	0.00	0.00	0.02	0.00	0.01
3	0.20	0.00	0.00	0.01	0.00	0.11	0.00	0.10
4	0.10	0.20	0.00	0.00	0.00	0.11	0.08	0.22
6	0.10	0.12	0.09	0.02	0.00	0.10	0.09	0.20
7	0.00	0.00	0.00	0.00	0.00	0.00	0.00	0.11
8	0.00	0.00	0.00	0.00	0.00	0.10	0.00	0.11
9	0.01	0.00	0.00	0.00	0.00	0.00	0.00	0.00

Table S14c: TRP DRTE>0.093 INDEX BI: Arrows

	1	2	3	4	6	7	8	9
1	0.00	0.00	0.00	0.00	0.00	0.20	0.09	0.00
2	0.00	0.00	0.00	0.00	0.00	0.00	0.00	0.00
3	0.20	0.00	0.00	0.00	0.00	0.11	0.00	0.10
4	0.10	0.20	0.00	0.00	0.00	0.11	0.00	0.22
6	0.10	0.12	0.00	0.00	0.00	0.10	0.00	0.20
7	0.00	0.00	0.00	0.00	0.00	0.00	0.00	0.11
8	0.00	0.00	0.00	0.00	0.00	0.10	0.00	0.11
9	0.00	0.00	0.00	0.00	0.00	0.00	0.00	0.00

Ticino River Park (Renyi Transfer Entropy) Day: H q=0.1

Table S15a: TRP RTE INDEX H: DAY

	1	2	3	4	6	7	8	9
1	0.00	0.11	0.11	0.11	0.39	0.12	0.12	0.15
2	0.12	0.00	0.11	0.17	0.24	0.11	0.11	0.09
3	0.11	0.10	0.00	0.18	0.24	0.11	0.11	0.21
4	-0.04	0.03	0.03	0.00	0.31	0.04	0.04	0.22
6	0.18	0.10	0.03	0.24	0.00	0.03	0.03	0.14
7	0.11	0.10	0.11	0.18	0.17	0.00	0.12	0.21
8	0.12	0.11	0.11	0.12	0.24	0.12	0.00	0.15
9	0.12	0.17	0.05	0.20	0.27	0.18	0.06	0.00

Table S15b: TRP DRTE INDEX H <DRTE> =0.078

	1	2	3	4	6	7	8	9
1	0.00	0.00	0.00	0.15	0.21	0.00	0.00	0.03
2	0.00	0.00	0.00	0.15	0.14	0.01	0.01	0.00
3	0.00	0.00	0.00	0.15	0.21	0.00	0.00	0.16
4	0.00	0.00	0.00	0.00	0.06	0.00	0.00	0.02
6	0.00	0.00	0.00	0.00	0.00	0.00	0.00	0.00
7	0.00	0.00	0.00	0.14	0.14	0.00	0.00	0.02
8	0.00	0.00	0.00	0.08	0.21	0.00	0.00	0.09
9	0.00	0.08	0.00	0.00	0.12	0.00	0.00	0.00

Table S15c: TRP DRTE>0.078 INDEX H: Arrows

	1	2	3	4	6	7	8	9
1	0.00	0.00	0.00	0.15	0.21	0.00	0.00	0.00
2	0.00	0.00	0.00	0.15	0.14	0.00	0.00	0.00
3	0.00	0.00	0.00	0.15	0.21	0.00	0.00	0.16
4	0.00	0.00	0.00	0.00	0.00	0.00	0.00	0.00
6	0.00	0.00	0.00	0.00	0.00	0.00	0.00	0.00
7	0.00	0.00	0.00	0.14	0.14	0.00	0.00	0.00
8	0.00	0.00	0.00	0.08	0.21	0.00	0.00	0.09
9	0.00	0.08	0.00	0.00	0.12	0.00	0.00	0.00

Ticino River Park (Renyi Transfer Entropy) Day: BI q=0.1

Table S16a: TRP RTE INDEX BI: DAY

	1	2	3	4	6	7	8	9
1	0.00	0.05	0.20	0.14	0.17	0.33	0.21	0.32
2	0.04	0.00	0.05	0.20	0.20	0.20	0.20	0.33
3	0.19	0.05	0.00	0.17	0.19	0.18	0.10	0.38
4	0.19	0.19	0.18	0.00	0.28	0.19	0.19	0.30
6	-0.03	0.05	0.19	0.08	0.00	0.27	0.07	0.32
7	0.11	0.11	0.18	0.12	0.25	0.00	0.04	0.23
8	0.19	0.18	0.04	0.19	0.26	0.11	0.00	0.31
9	0.12	0.05	0.26	0.19	0.20	0.11	0.24	0.00

Table S16b: TRP DRTE INDEX BI <DRTE> =0.086

	1	2	3	4	6	7	8	9
1	0.00	0.01	0.01	0.00	0.20	0.21	0.02	0.20
2	0.00	0.00	0.00	0.01	0.15	0.08	0.02	0.28
3	0.00	0.00	0.00	0.00	0.01	0.00	0.07	0.12
4	0.04	0.00	0.01	0.00	0.19	0.07	0.01	0.11
6	0.00	0.00	0.00	0.00	0.00	0.02	0.00	0.12
7	0.00	0.00	0.00	0.00	0.00	0.00	0.00	0.12
8	0.00	0.00	0.00	0.00	0.19	0.07	0.00	0.07
9	0.00	0.00	0.00	0.00	0.00	0.00	0.00	0.00

Table S16c: TRP DRTE>0.086 INDEX BI: Arrows

	1	2	3	4	6	7	8	9
1	0.00	0.00	0.00	0.00	0.20	0.21	0.00	0.20
2	0.00	0.00	0.00	0.00	0.15	0.00	0.00	0.28
3	0.00	0.00	0.00	0.00	0.00	0.00	0.00	0.12
4	0.00	0.00	0.00	0.00	0.19	0.00	0.00	0.11
6	0.00	0.00	0.00	0.00	0.00	0.00	0.00	0.12
7	0.00	0.00	0.00	0.00	0.00	0.00	0.00	0.12
8	0.00	0.00	0.00	0.00	0.19	0.00	0.00	0.00
9	0.00	0.00	0.00	0.00	0.00	0.00	0.00	0.00

Ticino River Park (Renyi Transfer Entropy) Night: H q=0.1

Table S17a: TRP RTE INDEX H: NIGHT

	1	2	3	4	6	7	8	9
1	0.00	-0.03	0.05	-0.03	0.12	0.26	0.11	0.13
2	0.11	0.00	0.19	0.12	0.11	0.25	0.11	-0.01
3	0.19	0.11	0.00	0.04	0.12	0.18	0.12	0.24
4	0.11	0.18	0.13	0.00	0.11	0.24	0.11	0.25
6	0.26	0.11	0.20	0.03	0.00	0.24	0.18	0.12
7	0.05	0.04	0.04	0.12	0.04	0.00	0.12	-0.01
8	0.12	0.04	0.13	0.04	0.11	0.23	0.00	-0.01
9	0.32	0.05	0.32	0.18	0.06	0.26	-0.02	0.00

Table S17b: TRP DRTE INDEX H <DRTE> =0.104

	1	2	3	4	6	7	8	9
1	0.00	0.00	0.00	0.00	0.00	0.21	0.00	0.00
2	0.15	0.00	0.08	0.00	0.00	0.22	0.08	0.00
3	0.14	0.00	0.00	0.00	0.00	0.13	0.00	0.00
4	0.14	0.06	0.08	0.00	0.08	0.12	0.07	0.07
6	0.14	0.00	0.09	0.00	0.00	0.20	0.06	0.06
7	0.00	0.00	0.00	0.00	0.00	0.00	0.00	0.00
8	0.01	0.00	0.01	0.00	0.00	0.11	0.00	0.01
9	0.19	0.06	0.08	0.00	0.00	0.27	0.00	0.00

Table S17c: TRP DRTE>0.104 INDEX H: Arrows

	1	2	3	4	6	7	8	9
1	0.00	0.00	0.00	0.00	0.00	0.21	0.00	0.00
2	0.15	0.00	0.00	0.00	0.00	0.22	0.00	0.00
3	0.14	0.00	0.00	0.00	0.00	0.13	0.00	0.00
4	0.14	0.00	0.00	0.00	0.00	0.12	0.00	0.00
6	0.14	0.00	0.00	0.00	0.00	0.20	0.00	0.00
7	0.00	0.00	0.00	0.00	0.00	0.00	0.00	0.00
8	0.00	0.00	0.00	0.00	0.00	0.11	0.00	0.00
9	0.19	0.00	0.00	0.00	0.00	0.27	0.00	0.00

Ticino River Park (Renyi Transfer Entropy) Night: BI q=0.1

Table S18a: TRP RTE INDEX BI: NIGHT

	1	2	3	4	6	7	8	9
1	0.00	0.11	0.13	0.10	0.18	0.19	0.24	0.34
2	0.10	0.00	0.18	0.11	0.11	0.26	0.11	0.20
3	0.20	0.19	0.00	0.10	0.12	0.25	0.18	0.17
4	0.25	0.11	0.05	0.00	0.18	0.04	0.11	0.21
6	0.18	0.18	0.12	0.18	0.00	0.19	0.11	0.25
7	0.26	0.34	0.18	0.03	0.12	0.00	0.05	0.25
8	0.46	0.26	0.24	0.24	0.04	0.10	0.00	0.19
9	0.46	0.26	0.11	0.19	0.18	0.21	0.12	0.00

Table S18b: TRP DRTE INDEX BI <DRTE> =0.065

	1	2	3	4	6	7	8	9
1	0.00	0.01	0.00	0.00	0.00	0.00	0.00	0.00
2	0.00	0.00	0.00	0.00	0.00	0.00	0.00	0.00
3	0.07	0.02	0.00	0.06	0.00	0.07	0.00	0.07
4	0.14	0.00	0.00	0.00	0.00	0.01	0.00	0.01
6	0.00	0.07	0.00	0.00	0.00	0.08	0.07	0.08
7	0.07	0.08	0.00	0.00	0.00	0.00	0.00	0.05
8	0.22	0.16	0.07	0.13	0.00	0.06	0.00	0.07
9	0.12	0.06	0.00	0.00	0.00	0.00	0.00	0.00

Table S18c: TRP DRTE>0.065 INDEX BI: Arrows

	1	2	3	4	6	7	8	9
1	0.00	0.00	0.00	0.00	0.00	0.00	0.00	0.00
2	0.00	0.00	0.00	0.00	0.00	0.00	0.00	0.00
3	0.07	0.00	0.00	0.00	0.00	0.07	0.00	0.07
4	0.14	0.00	0.00	0.00	0.00	0.00	0.00	0.00
6	0.00	0.07	0.00	0.00	0.00	0.08	0.07	0.08
7	0.07	0.08	0.00	0.00	0.00	0.00	0.00	0.00
8	0.22	0.16	0.07	0.13	0.00	0.00	0.00	0.07
9	0.12	0.00	0.00	0.00	0.00	0.00	0.00	0.00

Ticino River Park (Renyi Transfer Entropy) Dawn: H q=0.5

Table S19a: TRP RTE INDEX H: DAWN

	1	2	3	4	6	7	8	9
1	0.00	0.03	0.04	0.03	0.06	0.05	0.02	0.15
2	0.05	0.00	0.02	0.03	0.07	0.11	0.02	0.15
3	0.01	0.03	0.00	0.05	0.08	0.16	0.04	0.10
4	0.05	0.02	0.01	0.00	0.07	0.10	0.10	0.10
6	0.06	0.01	0.06	0.02	0.00	0.06	0.11	0.15
7	0.05	0.08	0.10	0.04	0.07	0.00	0.02	0.14
8	-0.03	0.02	0.01	-0.01	0.07	-0.02	0.00	0.18
9	0.10	0.11	0.10	0.09	0.11	0.05	0.04	0.00

Table S19b: TRP DRTE INDEX H <DRTE> =0.037

	1	2	3	4	6	7	8	9
1	0.00	0.00	0.03	0.00	0.00	0.00	0.04	0.05
2	0.02	0.00	0.00	0.01	0.06	0.03	0.00	0.04
3	0.00	0.01	0.00	0.03	0.02	0.06	0.03	0.00
4	0.01	0.00	0.00	0.00	0.05	0.06	0.11	0.00
6	0.00	0.00	0.00	0.00	0.00	0.00	0.04	0.04
7	0.00	0.00	0.00	0.00	0.00	0.00	0.04	0.09
8	0.00	0.00	0.00	0.00	0.00	0.00	0.00	0.14
9	0.00	0.00	0.01	0.00	0.00	0.00	0.00	0.00

Table S19c: TRP DRTE>0.037 INDEX H: Arrows

	1	2	3	4	6	7	8	9
1	0.00	0.00	0.00	0.00	0.00	0.00	0.04	0.05
2	0.00	0.00	0.00	0.00	0.06	0.00	0.00	0.00
3	0.00	0.00	0.00	0.00	0.00	0.06	0.00	0.00
4	0.00	0.00	0.00	0.00	0.05	0.06	0.11	0.00
6	0.00	0.00	0.00	0.00	0.00	0.00	0.04	0.04
7	0.00	0.00	0.00	0.00	0.00	0.00	0.04	0.09
8	0.00	0.00	0.00	0.00	0.00	0.00	0.00	0.14
9	0.00	0.00	0.00	0.00	0.00	0.00	0.00	0.00

Ticino River Park (Renyi Transfer Entropy) Dawn: BI q=0.5

Table S20a: TRP RTE INDEX BI: DAWN

	1	2	3	4	6	7	8	9
1	0.00	0.09	0.08	0.16	0.05	0.17	0.16	0.12
2	0.13	0.00	0.09	0.15	0.07	0.17	0.11	0.12
3	0.19	0.08	0.00	0.17	0.06	0.19	0.13	0.16
4	0.21	0.22	0.16	0.00	0.13	0.22	0.13	0.21
6	0.14	0.18	0.10	0.18	0.00	0.18	0.15	0.13
7	0.07	0.12	0.13	0.14	0.11	0.00	0.13	0.16
8	0.12	0.10	0.13	0.10	0.11	0.17	0.00	0.14
9	0.13	0.11	0.12	0.11	0.05	0.11	0.09	0.00

Table S20b: TRP DRTE INDEX BI <DRTE> =0.051

	1	2	3	4	6	7	8	9
1	0.00	0.00	0.00	0.00	0.00	0.10	0.04	0.00
2	0.04	0.00	0.01	0.00	0.00	0.05	0.01	0.01
3	0.10	0.00	0.00	0.01	0.00	0.06	0.00	0.04
4	0.05	0.07	0.00	0.00	0.00	0.08	0.04	0.11
6	0.08	0.11	0.04	0.06	0.00	0.07	0.04	0.08
7	0.00	0.00	0.00	0.00	0.00	0.00	0.00	0.06
8	0.00	0.00	0.00	0.00	0.00	0.04	0.00	0.05
9	0.01	0.00	0.00	0.00	0.00	0.00	0.00	0.00

Table S20c: TRP DRTE>0.051 INDEX BI: Arrows

	1	2	3	4	6	7	8	9
1	0.00	0.00	0.00	0.00	0.00	0.10	0.00	0.00
2	0.00	0.00	0.00	0.00	0.00	0.00	0.00	0.00
3	0.10	0.00	0.00	0.00	0.00	0.06	0.00	0.00
4	0.00	0.07	0.00	0.00	0.00	0.08	0.00	0.11
6	0.08	0.11	0.00	0.06	0.00	0.07	0.00	0.08
7	0.00	0.00	0.00	0.00	0.00	0.00	0.00	0.06
8	0.00	0.00	0.00	0.00	0.00	0.00	0.00	0.00
9	0.00	0.00	0.00	0.00	0.00	0.00	0.00	0.00

Ticino River Park (Renyi Transfer Entropy) Day: H q=0.5

Table S21a: TRP RTE INDEX H: DAY

	1	2	3	4	6	7	8	9
1	0.00	0.03	0.04	0.03	0.11	0.05	0.07	0.04
2	0.05	0.00	0.03	0.04	0.05	0.04	0.04	0.02
3	0.05	0.02	0.00	0.05	0.06	0.03	0.04	0.06
4	-0.02	-0.00	0.01	0.00	0.05	0.02	0.01	0.08
6	0.06	0.02	-0.00	0.04	0.00	-0.00	0.00	0.06
7	0.05	0.03	0.03	0.04	0.02	0.00	0.04	0.07
8	0.07	0.03	0.03	0.04	0.05	0.04	0.00	0.05
9	0.04	0.03	0.00	0.05	0.08	0.06	0.03	0.00

Table S21b: TRP DRTE INDEX H <DRTE> =0.022

	1	2	3	4	6	7	8	9
1	0.00	0.00	0.00	0.05	0.05	0.00	0.00	0.00
2	0.02	0.00	0.01	0.04	0.03	0.01	0.01	0.00
3	0.01	0.00	0.00	0.04	0.06	0.00	0.01	0.06
4	0.00	0.00	0.00	0.00	0.00	0.00	0.00	0.03
6	0.00	0.00	0.00	0.00	0.00	0.00	0.00	0.00
7	0.00	0.00	0.00	0.02	0.02	0.00	0.00	0.01
8	0.00	0.00	0.00	0.02	0.05	0.00	0.00	0.02
9	0.00	0.00	0.00	0.00	0.02	0.00	0.00	0.00

Table S21c: TRP DRTE>0.022 INDEX H: Arrows

	1	2	3	4	6	7	8	9
1	0.00	0.00	0.00	0.05	0.05	0.00	0.00	0.00
2	0.00	0.00	0.00	0.04	0.03	0.00	0.00	0.00
3	0.00	0.00	0.00	0.04	0.06	0.00	0.00	0.06
4	0.00	0.00	0.00	0.00	0.00	0.00	0.00	0.03
6	0.00	0.00	0.00	0.00	0.00	0.00	0.00	0.00
7	0.00	0.00	0.00	0.02	0.00	0.00	0.00	0.00
8	0.00	0.00	0.00	0.02	0.05	0.00	0.00	0.00
9	0.00	0.00	0.00	0.00	0.00	0.00	0.00	0.00

Ticino River Park (Renyi Transfer Entropy) Day: BI q=0.5

Table S22a: TRP RTE INDEX BI: DAY

	1	2	3	4	6	7	8	9
1	0.00	0.03	0.08	0.03	0.04	0.10	0.07	0.06
2	0.01	0.00	0.02	0.06	0.07	0.07	0.06	0.10
3	0.07	0.04	0.00	0.05	0.05	0.03	0.02	0.08
4	0.06	0.08	0.07	0.00	0.07	0.07	0.04	0.04
6	0.01	0.04	0.04	0.00	0.00	0.11	0.03	0.07
7	0.03	0.04	0.04	0.05	0.05	0.00	0.00	0.05
8	0.07	0.06	0.00	0.03	0.07	0.03	0.00	0.05
9	0.05	0.02	0.07	0.05	0.06	0.04	0.04	0.00

Table S22b: TRP DRTE INDEX BI <DRTE> =0.025

	1	2	3	4	6	7	8	9
1	0.00	0.02	0.01	0.00	0.03	0.07	0.00	0.01
2	0.00	0.00	0.00	0.00	0.03	0.03	0.01	0.07
3	0.00	0.02	0.00	0.00	0.01	0.00	0.02	0.02
4	0.03	0.02	0.02	0.00	0.07	0.02	0.00	0.00
6	0.00	0.00	0.00	0.00	0.00	0.06	0.00	0.01
7	0.00	0.00	0.01	0.00	0.00	0.00	0.00	0.01
8	0.00	0.00	0.00	0.00	0.04	0.03	0.00	0.01
9	0.00	0.00	0.00	0.01	0.00	0.00	0.00	0.00

Table S22c: TRP DRTE>0.025 INDEX BI: Arrows

	1	2	3	4	6	7	8	9
1	0.00	0.00	0.00	0.00	0.03	0.07	0.00	0.00
2	0.00	0.00	0.00	0.00	0.03	0.03	0.00	0.07
3	0.00	0.00	0.00	0.00	0.00	0.00	0.00	0.00
4	0.03	0.00	0.00	0.00	0.07	0.00	0.00	0.00
6	0.00	0.00	0.00	0.00	0.00	0.06	0.00	0.00
7	0.00	0.00	0.00	0.00	0.00	0.00	0.00	0.00
8	0.00	0.00	0.00	0.00	0.04	0.03	0.00	0.00
9	0.00	0.00	0.00	0.00	0.00	0.00	0.00	0.00

Ticino River Park (Renyi Transfer Entropy) Night: H q=0.5

Table S23a: TRP RTE INDEX H: NIGHT

	1	2	3	4	6	7	8	9
1	0.00	-0.00	0.03	-0.01	0.06	0.09	0.04	0.07
2	0.03	0.00	0.06	0.04	0.04	0.08	0.04	0.00
3	0.06	0.03	0.00	0.03	0.05	0.05	0.05	0.08
4	0.02	0.05	0.06	0.00	0.04	0.06	0.03	0.09
6	0.08	0.03	0.08	0.01	0.00	0.06	0.04	0.04
7	0.02	0.02	0.03	0.06	0.03	0.00	0.05	0.02
8	0.02	0.01	0.06	0.02	0.02	0.04	0.00	-0.00
9	0.09	0.02	0.11	0.07	0.04	0.09	-0.02	0.00

Table S23b: TRP DRTE INDEX H <DRTE> =0.026

	1	2	3	4	6	7	8	9
1	0.00	0.00	0.00	0.00	0.00	0.07	0.02	0.00
2	0.03	0.00	0.02	0.00	0.00	0.06	0.03	0.00
3	0.03	0.00	0.00	0.00	0.00	0.02	0.00	0.00
4	0.03	0.01	0.03	0.00	0.03	0.00	0.02	0.02
6	0.02	0.00	0.04	0.00	0.00	0.04	0.02	0.01
7	0.00	0.00	0.00	0.00	0.00	0.00	0.01	0.00
8	0.00	0.00	0.01	0.00	0.00	0.00	0.00	0.02
9	0.03	0.02	0.04	0.00	0.00	0.07	0.00	0.00

Table S23c: TRP DRTE>0.026 INDEX H: Arrows

	1	2	3	4	6	7	8	9
1	0.00	0.00	0.00	0.00	0.00	0.07	0.00	0.00
2	0.03	0.00	0.00	0.00	0.00	0.06	0.03	0.00
3	0.03	0.00	0.00	0.00	0.00	0.00	0.00	0.00
4	0.00	0.00	0.03	0.00	0.03	0.00	0.00	0.00
6	0.00	0.00	0.04	0.00	0.00	0.04	0.00	0.00
7	0.00	0.00	0.00	0.00	0.00	0.00	0.00	0.00
8	0.00	0.00	0.00	0.00	0.00	0.00	0.00	0.00
9	0.00	0.00	0.04	0.00	0.00	0.07	0.00	0.00

Ticino River Park (Renyi Transfer Entropy) Night: BI q=0.5

Table S24a: TRP RTE INDEX BI: NIGHT

	1	2	3	4	6	7	8	9
1	0.00	0.02	0.07	0.01	0.05	0.06	0.07	0.12
2	0.01	0.00	0.05	0.03	0.05	0.07	0.03	0.09
3	0.05	0.03	0.00	0.01	0.04	0.05	0.04	0.04
4	0.04	0.02	0.01	0.00	0.05	0.02	0.03	0.10
6	0.02	0.05	0.04	0.05	0.00	0.06	0.04	0.09
7	0.07	0.11	0.05	0.01	0.04	0.00	0.03	0.08
8	0.14	0.08	0.07	0.06	0.02	0.02	0.00	0.08
9	0.14	0.09	0.01	0.07	0.05	0.06	0.05	0.00

Table S24b: TRP DRTE INDEX BI <DRTE> =0.021

	1	2	3	4	6	7	8	9
1	0.00	0.01	0.02	0.00	0.03	0.00	0.00	0.00
2	0.00	0.00	0.02	0.01	0.00	0.00	0.00	0.01
3	0.00	0.00	0.00	0.00	0.00	0.00	0.00	0.03
4	0.03	0.00	0.00	0.00	0.01	0.01	0.00	0.03
6	0.00	0.00	0.00	0.00	0.00	0.02	0.02	0.04
7	0.01	0.04	0.00	0.00	0.00	0.00	0.01	0.02
8	0.08	0.05	0.02	0.03	0.00	0.00	0.00	0.03
9	0.03	0.00	0.00	0.00	0.00	0.00	0.00	0.00

Table S24c: TRP DRTE>0.021 INDEX BI: Arrows

	1	2	3	4	6	7	8	9
1	0.00	0.00	0.00	0.00	0.03	0.00	0.00	0.00
2	0.00	0.00	0.00	0.00	0.00	0.00	0.00	0.00
3	0.00	0.00	0.00	0.00	0.00	0.00	0.00	0.03
4	0.03	0.00	0.00	0.00	0.00	0.00	0.00	0.03
6	0.00	0.00	0.00	0.00	0.00	0.00	0.00	0.04
7	0.00	0.04	0.00	0.00	0.00	0.00	0.00	0.00
8	0.08	0.05	0.02	0.03	0.00	0.00	0.00	0.03
9	0.03	0.00	0.00	0.00	0.00	0.00	0.00	0.00
